# Ferritin nanocage-enabled detection of pathological tau in living human retinal cells

**DOI:** 10.1038/s41598-024-62188-8

**Published:** 2024-05-21

**Authors:** Lorenzo Barolo, Ylenia Gigante, Lorenza Mautone, Silvia Ghirga, Alessandro Soloperto, Alessandra Giorgi, Francesca Ghirga, Martina Pitea, Alessio Incocciati, Francesco Mura, Giancarlo Ruocco, Alberto Boffi, Paola Baiocco, Silvia Di Angelantonio

**Affiliations:** 1 Department of Biochemical Sciences, Sapienza University of Rome, 00185 Rome, Italy; 2 Center for Life Nano- and Neuro-Science, Istituto Italiano di Tecnologia, 00161 Rome, Italy; 3 D-Tails Srl BC, 00165 Rome, Italy; 4 Department of Physiology and Pharmacology, Sapienza University of Rome, 00185 Rome, Italy; 5 Department of Chemistry and Technology of Drugs, Sapienza-University of Rome, 00185 Rome, Italy; 6 Research Center on Nanotechnologies Applied to Engineering of Sapienza (CNIS), Sapienza University of Rome, 00185 Rome, Italy; 7 Department of Physics, Sapienza University of Rome, 00185 Rome, Italy

**Keywords:** Tauopathies, Alzheimer’s disease, Frontotemporal Dementia, Nanobiotechnology, Ferritin nanocages, BODYPY tau fluorophores, Neurodegenerative diseases, Diagnostic probes, Nanomedicine, Induced pluripotent stem cells, Nanoscale delivery systems, Retinal tissue detection, Alzheimer's disease, Diagnostics

## Abstract

Tauopathies, including Alzheimer’s disease and Frontotemporal Dementia, are debilitating neurodegenerative disorders marked by cognitive decline. Despite extensive research, achieving effective treatments and significant symptom management remains challenging. Accurate diagnosis is crucial for developing effective therapeutic strategies, with hyperphosphorylated protein units and tau oligomers serving as reliable biomarkers for these conditions. This study introduces a novel approach using nanotechnology to enhance the diagnostic process for tauopathies. We developed humanized ferritin nanocages, a novel nanoscale delivery system, designed to encapsulate and transport a tau-specific fluorophore, BT1, into human retinal cells for detecting neurofibrillary tangles in retinal tissue, a key marker of tauopathies. The delivery of BT1 into living cells was successfully achieved through these nanocages, demonstrating efficient encapsulation and delivery into retinal cells derived from human induced pluripotent stem cells. Our experiments confirmed the colocalization of BT1 with pathological forms of tau in living retinal cells, highlighting the method’s potential in identifying tauopathies. Using ferritin nanocages for BT1 delivery represents a significant contribution to nanobiotechnology, particularly in neurodegenerative disease diagnostics. This method offers a promising tool for the early detection of tau tangles in retinal tissue, with significant implications for improving the diagnosis and management of tauopathies. This study exemplifies the integration of nanotechnology with biomedical science, expanding the frontiers of nanomedicine and diagnostic techniques.

## Introduction

The recent development of tau specific in vivo staging of Alzheimer’s disease (AD), and more in general of tau pathologies such as Frontotemporal Dementia (FTD), is a compelling target in clinical research in order to track disease changes longitudinally and in the continuum relation to established biomarkers. There are currently about 200 active clinical trials worldwide assessing 140 novel treatments for AD, requiring a total enrollment of more than 60,000 participants^1–3^. In all clinical trials on AD, inclusion criteria from patient enrollment to concluding evaluation at the end of the study, require careful evaluation of suitable biomarkers^4^. MRI is the most common entry-level analysis collected as an outcome measure, followed by amyloid PET and tau PET^4–6^. The current trend for novel clinical trials, however, increasingly requires advances in ultra-sensitive methods to detect proteins in cerebrospinal fluid (CSF) and plasma that enable the identification of pathological changes along the AD continuum. Despite the progress made in identifying plasma tau species that correlate with amyloid accumulation in early stages and neurodegeneration at later stages, current plasma assays have not yet been shown to detect specific tau species that reflect tau aggregation in the brain^7^. In this framework, markers that are able to directly correlate brain aggregates with clinical AD manifestation are long-awaited for the AD diagnostics and follow-up. In contrast to the brain, the retina is unique in that it can be visualized in vivo using optical imaging methods, such as fundus photography (FP), optical coherence tomography (OCT), OCT‐angiography (OCT‐A), fluorescence lifetime imaging ophthalmoscopy (FLIO), and hyperspectral imaging (HSI)^8–13^. Thus far, retinal imaging represents a window of opportunity to assess neurodegenerative AD, through the eye^13–16^. The fast, easy, cheap, and non-invasive characteristics of the method would be an invaluable help to speed up the monitoring of neurodegenerative disease progression in the continuum. In this context, retinal imaging, especially when coupled to a tau protein-sensitive fluorescent marker, is expected to revolutionize clinical trials on novel pharma products and become a routine for the screening even for asymptomatic, early-stage, AD, and FTD patients. Recent developments in fluorescent tau probes have enabled in vitro staging of tau pathology and improved our understanding of the pathogenesis of diseases in small-animal models fluorescence imaging^17–20^. In particular, BODIPY-derived fluorophores with excellent fluorescence properties in the visible and near-infrared regions have contributed to tau detection and represent the frontier of the research in the field^18,19,21–24^. BODIPY scaffold, modified by a conjugated aromatic system, appears to bind selectively and with high affinity to the hyperphosphorylated tau protein aggregates, the core component of neurofibrillary tangle pathology^22,25^. These unique properties match with the ability to discriminate tau fibrils from beta-amyloid (Aβ) and other β-sheet-structured protein aggregates^18,19,21–24^. Critical issues for the successful application of these compounds in vivo concern the low water solubility of BODIPY-based compounds together with appropriate delivery systems to the retinal cells. Most recently, fused heptacyclotriene-BODIPY scaffolds have been obtained to overcome solubility limitations while maintaining a high affinity for pathological forms of the tau protein and favorable optical properties^26,27^. In contrast, options for targeted delivery carriers have not yet been addressed. Moreover, the choice of the preclinical model to test tau ligands represents a challenging issue. Indeed, many potential drug candidates for therapeutic and diagnostic purposes fail during pre-clinical trials involving testing on mouse models, mainly because of the significant differences between rodents and humans. Consequently, in vitro humanized models of neurodegenerative diseases, based on human-induced Pluripotent Stem Cells (hiPSCs), represent valuable tools for screening potential diagnostic and therapeutic drugs^28–34^. In the present work, the fluorescent BODIPY (BT1)^21,22^ was entrapped into protein-based nanocages to improve the cell penetration ability and to enable a targeted uptake in human iPSC-derived retinal cells under physiological and pathological conditions. In particular, the delivery of BT1 was developed using the humanized *Archaeoglobus* ferritin (HumAfFt) characterized by an 8 nm internal diameter and capable of being uptaken by the CD71 receptor^35–39^. The encapsulation of hydrophobic compounds was accomplished through chemical crosslinking of the internal cavity addressing solubility issues through the establishment of hydrophobic interactions. Notably, the nanocage system demonstrates a high level of efficiency in entering human iPSC-derived retinal cells, ultimately facilitating the release of the fluorescent marker in close proximity to pathological tau forms.

## Methods

### BT1 solubility analysis

The fluorescent probe BT1 was synthesized as described in Soloperto et al.^22^ It was resuspended in DMSO 100% up to a concentration of 2 mM. The concentration of BT1 was determined via UV–Vis spectrophotometry using a calibration curve in the range of 10 to 200 μM of BT1 under the same experimental conditions using a linear correlation (R^2^ = 0.99) (Figure S1). To analyze the proportionality between concentration and fluorescence, the probe was diluted to various concentrations in DMSO (ranging from 90 to 2.5 µM). The fluorescence analysis was repeated for BT1 resuspended in a polar aqueous buffer such as Hepes 20 mM, pH 7.4. Various concentrations were analyzed in the range from 165 to 1 µM. All the spectra were measured in the range between 530 and 700 nm with excitation wavelength at 520 nm by using a Shimadzu Fluorimeter RF-6000.

### Recombinant HumAfFt preparation

HumAfFt was designed and genetically engineered to be recognized by the human TfR1 receptor and an M54C mutation per monomer was inserted to functionalize the protein inner cavity with thiol-reactive compounds. The HumAfFt was expressed and purified as described elsewhere^35^. Briefly, 10 g of harvested cells were resuspended in 50 mL of lysis buffer (20 Hepes, 300 mM NaCl, pH 7.4, and a cOmplete™ Mini Protease Inhibitor Cocktail Tablet (Roche)) DNAse and MgCl_2_ 5 mM. After disruption by sonication, the lysate was centrifuged at 10,000 rpm for 30 min at 4 °C. The supernatant was separated from the pellet and subsequently heated at 78 °C for 10 min to promote the precipitation of a large amount of undesired E. coli proteins. After heating, the sample was centrifuged at 10,000 rpm for 30 min at 4 °C. The supernatant was fractionated by ammonium sulfate precipitation. 70% ammonium sulfate pellet containing highly purified protein was resuspended in 20 mM Hepes, 50 mM MgCl_2_, inserted in dialysis tubing cellulose membrane, and left in a 2 L solution of the same dialysis buffer. The recombinant HumAfFt was purified using one-step chromatography. Size-exclusion chromatography (SEC) was performed, using a HiPrep™ 16/60 Sephacryl® S-400 (GE Healthcare) in 20 mM Hepes, 50 mM MgCl_2_, pH 7.4. Protein purification was confirmed via gel electrophoresis. The final concentration of HumAfFt was determined via UV–Vis spectrophotometry (Jasco-V750, Corp. Tokyo, Japan) using a molar extinction coefficient, ε_280nm_, for HumAfFt of 32,400 M^−1^ cm^−1^. The purification yield was about 150 mg/10 g of bacterial paste. The supernatant was collected and sterilized with a 0.22 µm filter and stored at 4 °C.

### Recombinant HumAfFt functionalization

Multiple N-substituted maleimide compounds bearing different functionalities, such as N-(1-Pyrenyl)maleimide (NPM), 6-maleimidohexanoic acid and 1,6-Bis(maleimido)hexane, were linked to react with the 24 cysteines inside the HumAfFt 24-mer.Hydrophobic maleimide such as N-(1-Pyrenyl)maleimide (NPM) was resuspended in DMSO 100%, reaching a final concentration of 10 mM. A starting concentration of 96 µM of HumAfFt monomers (4 µM in 24-mer) was chosen. Before the insertion of the maleimide, the cysteines of the HumAfFt were reduced by adding 960 µM (10 × the concentration of monomers) of tris(2-carboxyethyl)phosphine hydrochloride (TCEP) to the sample. The reaction was carried at 55 °C for 30 min. Then, 480 µM of maleimide (5 × the concentration of monomers) was added to the reduced HumAfFt. The reaction was carried at 30 °C for 2 h. At the end of this reaction, the unbounded maleimide was removed using a PD-10 desalting column equilibrated in Hepes 20 mM, pH 7.4 buffer to keep HumAfFt in the open conformation.

### BT1 encapsulation in NPM-HumAfFt complex

The BT1 probe (40 µM) was added to the functionalized NPM-HumAfFt (4 µM) in Hepes 20 mM. The sample was kept overnight at RT. At the end of the reaction, a final concentration of 50 mM of MgCl_2_ was added to the protein sample, to close the HumAfFt as 24-mer, creating the internal cavity. The unbound BT1 was removed through double centrifugation at 10,000 rpm for 30 min and filtration using a 0.22 µm filter. The sample was kept sterile at 4° C. Absorbance and fluorescence spectra were collected before and after the unbound probe removal to assess the probe incorporation. The BT1-loaded NPM-HumAfFt was analyzed through Dynamic Light Scattering (DLS) experiments using a Zetasizer Nano S (Malvern Instruments, Malvern, UK) equipped with a 4 mW He–Ne laser (633 nm). The measurements were performed at 25 °C, at an angle of 173° to the incident beam. The average hydrodynamic diameters (Z-average diameter) of the scattering particles were calculated using peak intensity analyses. Samples were prepared at 1 mg/mL in 20 mM Hepes, 50 mM MgCl_2_, pH 7.4.

### HPLC analysis

The protein purity and aggregation state before and after the complex formation were analyzed by high-performance size exclusion chromatography (HP-SEC) with an Agilent Infinity 1260 HPLC apparatus equipped with UV detector. The separation employed an Agilent AdvanceBio SEC 300 Å 2.7 µm 4.6 × 150 mm column, with 20 mM Hepes buffer (pH 7.4) containing 50 mM MgCl_2_ as the mobile phase. The flow rate was 0.7 mL/min over an elution window of 10 min. Ferritin elution was monitored by UV detection at 280 nm.

### Scanning transmission electron mycroscopy

The analysis of the size and shape of the nanoparticles after encapsulation of BT1, was performed using a Zeiss Auriga Transmission Electron Microscope in Scanning Mode (STEM). All the samples were prepared at 0.5 mg/mL. A single drop of each sample was applied onto a carbon-coated 3 mm copper grid, allowing for adsorption over 5 min. Subsequently, the grid was rinsed with a single drop of water for 5 min, followed by the addition of uranyl acetate staining solution (3%), left for 5 min, and then washed twice with H_2_O for 5 min each time.

### Spectrophotometric analysis

To determine the ratio of BT1 per nanoparticle, spectroscopic analysis was performed using a UV–Vis Jasco-V750 spectrophotometer (Jasco Corp. Tokyo, Japan). BT1-loaded NPM-HumAfFt samples were dissolved in 100% DMSO with 150 mM EDTA to induce opening of the nanoparticles and the concentration of the BT1 released was calculated by absorbance measurements using a calibration curve in the range of 10 to 200 μM of BT1 under the same experimental conditions using a linear correlation resulting in R^2^ = 0.999 (Figure S1).

### Detection of K18-Tau aggregates using the BT1-loaded NPM-HumAfFt

The K18 domain of Tau protein used in this study comprised the four repeat domains of the isoform hTau40 from residues 244 to 372. The K18 domain was purified as described in Soloperto et al.^22^ K18 tau protein was diluted in PBS to a final concentration of 75 µM. The formation of fibrils was achieved by reduction with 10 eq. TCEP at 55 °C for 10 min and by the addition of the promoter aggregation agent such as heparin in a ratio 1:1 = heparin:protein (75 µM) at 37 °C. A sample of K18 tau protein was not fibrillated and used as a control. To validate the target specificity of BT1, a model protein was selected to be fibrillated and analyzed using the same method. It has been reported that bovine serum albumin (BSA) generates fibrils after exposure at 63 °C within 5 h^22,40,41^. Therefore, BSA was diluted in PBS to a final concentration of 75 µM and subjected to 63 °C. A sample of BSA protein was not fibrillated and used as a control. Before performing the fluorescence experiment using the BT1-loaded NPM-HumAfFt, the formation of fibrils of both K18 and BSA was confirmed using a well-known fluorophore, thioflavin T (ThT) (Figure S2). Given these results, the fibrillation time points for K18 and BSA were selected. K18 tau protein with heparin was fibrillated at 37 °C for 1, 3, 4, 5, 6, 7 and 14 days. BSA protein was fibrillated at 63 °C for 0.5, 1, 3, and 5 h. Subsequently, the BT1 dye entrapped in the NPM-HumAfFt nanocage was added to the solutions to a final concentration of HumAfFt of 1 µM. The fluorescence emission of the samples was monitored at an excitation wavelength of 520 nm and emission at 565 nm by using a Shimadzu Fluorimeter RF-6000 (Shimadzu, Italia S.R.L.). The relative fluorescence was calculated based on the non-fibrillated control. The emission value at 565 of the control mixed with the BT1 dye in NPM-HumAfFt was considered as 10 arbitrary units (a.u.), and the values of the other samples were calculated accordingly.

### Retinal culture differentiation from human iPSC

Retinal cultures were differentiated from healthy (SIGi001-A, EBiSC/Sigma) and tau-mutant human induced-pluripotent stem cell lines (SIGi001-A-13, EBiSC/Sigma) according to a previously established protocol^42,43^ with minor modifications. Human iPSCs were dissociated to single cells with Accutase (Gibco) and were resuspended in mTeSR Plus with 10 µM Y-27362 (Stemcell). Dissociated hiPSC were plated on growth factor reduced Matrigel coated dishes (Corning, dilution 1:100) at a density of 1000 cells/mm2. The day of seeding was defined as day in vitro (DIV) -2. Next day, medium was completely replaced with neurogenic basal medium composed of 50% DMEM/F12 (Sigma), 50% Neurobasal (ThermoFisher), 1% GlutaMAX Supplement (Gibco), 0.1% Pen-Strep (Sigma), 1% NEAA (Gibco), 1% N2 Supplement (ThermoFisher), and 2% B27 Supplement w/oA (Gibco). The medium was refreshed daily for 10 days, and every 2 days until DIV 13, when retinal progenitor islands appear in culture. Afterward, the islands were dissociated with Dispase II (Millipore Corporate) and the pellet was resuspended in 1 ml of neurogenic basal medium. Thereafter, 300 µl of suspension were plated onto PLO/Laminin (Sigma) coated petri dish (60 mm). Reaching DIV 20, retinal progenitors were dissociated with Accutase and plated onto PLO/Laminin coated petri dish at the density of 1000/mm2 until DIV 30 for RNA extraction. Instead, for immunostaining, live/dead and internalization analysis DIV 20 retinal neurons are plated onto a PLO/Laminin coated cover glasses circle (0.12 mm, ThorLab) at a density of 100.000 cells per glass until DIV 30. A different mix of small molecules was added to the medium at specific intervals: 1 µM Dorsomorphin (Sigma) and 2.5 µM IDE2 (Sigma) from DIV 0 to DIV 6; 10 mM Nicotinamide (Sigma) until DIV 10; 25 µM Forskolin (Sigma) from DIV 0 to DIV 30; 10 µM DAPT (Prepotech) from DIV 20 to DIV 30. To induce tau hyperphosphorylation, differentiated DIV 30 retinal neurons were treated with 100 µM of Okadaic acid (Merck Life Science) for 4 h at 37 °C in 5% CO_2_.

### RNA extraction and RT-qPCR

RNA samples were collected at DIV 0, 6, 20, 30 from 3 biological replicates of healthy and tau-mutant iPSC-derived neurons. Total RNA was extracted with EZNA Total RNA Kit I (Omega Bio-Tek). RNA concentration and purity were assessed spectrophotometrically by the ratio A260/280 values obtained by Nanodrop (Thermo Fisher). RNA samples were retro-transcribed using the iScript Reverse Transcription Supermix (Bio-Rad). RT-qPCR was performed with iTaq Universal SYBR Green Supermix (Bio-Rad) on a ViiA 7 Real-Time PCR System (Applied Biosystems) and gene expression data was reported as relative expression (2^−ΔΔCT^), normalized to internal control (GAPDH) and compared to control sample (DIV 0, undifferentiated iPSCs). Temporal gene expression toward retinal differentiation was carried out using the primers: GAPDH (FW: GGC CAT CCA CAG TCT TCT G, RV: TCA TCA GCA ATG CCT CCT G), POU4F1 (FW: ACCACCATTATTACCACCTCCC, RV: CTCGCTCGTTTGGTTTTCGTT), RCVRN (FW: TTCAAGGAGTACGTCATCGCC, RV: GATGGTCCCGTTACCGTCC), RLBP1 (FW: GGCAGGGAACAACCAAGACT, RV: AGTCAGGGCCAAGTTGTGAC), TFRC (FW: CGTGAGGCTGGATCTCAAA, RV: CCAGGATTCTCCACCAGGTA), NANOG (FW: GAAATACCTCAGCCTCCAGC, RV: GCGTCACACCATTGCTATTC).

### Immunostaining, image acquisition, and analysis of transferrin receptor expression in retinal cultures

Differentiated DIV 30 neurons were fixed with 4% paraformaldehyde (PFA, Sigma Aldrich) for 15 min at room temperature. For immunostaining, fixed retinal neurons were permeabilized with PBS containing 0.2% Triton X-100 (Sigma Aldrich) for 10 min and, to reduce non-specific binding of the primary antibody within the cell, then were incubated for 45 min at room temperature with a blocking solution containing PBS 0.1% Tween-20 (Sigma-Aldrich) and 5% goat serum (Merck KGaA, Darmstadt, Germany). Subsequently, cells were incubated overnight at 4 °C with primary antibodies. After washing out the primary antibody, the cells were incubated for 1 h at room temperature with secondary antibody Goat anti-rabbit, anti-chicken and anti-mouse Alexa Fluor Plus (1:750, ThermoFisher Scientific). Washes after primary and secondary antibody staining were performed with PBS solution containing 0.1% Tween-20, adding Hoechst (1:300, 33258, Merck) in the last wash to stain nuclei. In order to prepare the cells for analysis, round cover glasses with attached and stained cells were sealed with rectangular ones using the Prolong glass antifade mounting medium (P36984, Invitrogen). To test the specificity of staining, control experiments were performed in the absence of primary antibody incubation. For fluorescent image analysis, three independent batches of cultures for each condition were carried out.

Primary antibodies used: TUJ1 (1:1000 rabbit, T2200, Sigma), MAP2 (1:2000 chicken, ab5392, Abcam), BRN3a (1:50 mouse, sc-8429, Santa-CRuz); DM1A (1:500 mouse, T6199, Sigma), CD71 (1:50 rabbit, ab214039, Abcam); PAX6 (1.50 mouse, MA1-109, Invitrogen); GFAP (1:500 chicken, 173006 Synaptic system); TH (1:100 rabbit, ab152, Merck); RECOVERIN (1:500 rabbit, PA582541, invitrogen).

Confocal images of TUJ1, MAP2 and BRN3a staining were acquired with water objective 60X/NA 2.0 on Fluoview FV10i (Olympus) confocal laser scanning microscope.

Confocal images of CD71, DM1A (1:500 mouse, T6199, Sigma), were acquired with oil objective 60X/NA 1.42 (Olympus) on an Olympus iX73 microscope equipped with an X-Light V3 spinning disc head (CrestOptics), an LDI laser illuminator (89 North), a PRIME cMOS camera and a MetaMorph software (Molecular Devices).

Maximum Z-projections of fluorescence image stacks were analyzed using the MATLAB software. DM1A signals were used to delineate the region of interest, which was subsequently applied to the CD71 images for the quantification of puncta distributed along dendritic processes. The image analysis process comprised two main steps: DM1A segmentation and CD71 puncta quantification. Both images underwent preprocessing, including a Gaussian filter (sigma = 3 pixels) and background removal. In the first step, DM1A images were binarized using a global thresholding method. For the second step, the cytoskeletal mask derived from DM1A segmentation was applied to the maximum Z-projections of the CD71 image stack. Puncta detection was obtained using the findcircle function in MATLAB (with parameters: r = 1–10 pixels, threshold = 0.2 Otsu threshold). Lastly, the number of puncta within the region of interest was quantified and normalized based on the count of cells in each field of view.

### Analysis of BT1-loaded NPM-HumAfFt complex internalization into hiPSCs-derived retinal neurons

To assess the internalization, differentiated DIV 30 retinal neurons plated onto cover glasses were incubated with 150 nM of human AfFt-rhodamine for different hours (6–8–16–24–28) at 37 °C in 5% CO_2_. Subsequently, the cell culture medium was removed, and neurons were washed one time before adding the fresh stained solution (8 µg/ml of Fluorescein Diacetate, FDA and Hoechst 1:300). Cells were incubated at room temperature for 5 min in the dark. Then, neurons were washed once and round cover glasses were transferred into an Ibidi glass bottom dish in HEPES-buffered external solution (NES) containing 140 mM NaCl, 2.8 mM KCl, 2 mM CaCl_2_, 2 mM MgCl_2_, 10 mM HEPES, 10 mM D-glucose (pH 7.3 with NaOH; 290 mOsm). Live imaging was performed with an air objective 20X/NA 0.42 (Olympus) on an Olympus iX73 microscope equipped with an X-Light V3 spinning disc head (CrestOptics), an LDI laser illuminator (89 North), a PRIME cMOS camera and a MetaMorph software (Molecular Devices). Stack images were analyzed with the ImageJ software^44^, flattened in a maximum intensity Z-projection of 20 slices (z-step of 0.2 μm). Background noise was subtracted from stacked images and the integrated density was measured applying Triangle automatic thresholding method on ImageJ. Data was plotted in GraphPad Prism version 8.0 for Mac (GraphPad Software, San Diego, California USA, www.graphpad.com). The analysis was performed on 15/3/3 (FOV/batches/cover glasses) per condition and statistical significance was evaluated using one-way ANOVA.

### Cytotoxicity test of BT1-loaded NPM-HumAfFt complex

Toxicity of BT1 was evaluated using Fluorescence-based live-dead assays with fluorescein diacetate (FDA) and propidium iodide (PI), which stain viable cells and dead cells, respectively. The staining solution was freshly prepared with 8 µg/ml of FDA, 20 µg/ml of PI, and Hoechst (1:300) to stain nuclei. DIV 30 hiPSC-derived retinal neurons were incubated with different concentrations of AfFt-maleimide-BT1 (100 nM, 150 nM, 300 nM, 600 nM) for 24 h. Then, neurons were incubated with a staining solution at room temperature for 5 min in the dark. Round cover glasses with neurons were transferred into an Ibidi glass bottom dish in HEPES-buffered external solution (NES) containing 140 mM NaCl, 2.8 mM KCl, 2 mM CaCl_2_, 2 mM MgCl_2_, 10 mM HEPES, 10 mM D-glucose (pH 7.3 with NaOH; 290 mOsm). Live imaging was performed with an air objective 20X/NA 0.42 (Olympus) on an Olympus iX73 microscope equipped with an X-Light V3 spinning disc head (CrestOptics), an LDI laser illuminator (89 North), a PRIME cMOS camera and a MetaMorph software (Molecular Devices). The analysis was performed counting the total number of cells, the live cells in the green channel and the dead cells in the red channel, using the ImageJ Software. The percentage of live cells (Live Cells/Total Cells Number) * 100 and the percentage of dead cells (Dead Cells/Total Cells Number) * 100 was plotted in GraphPad Prism version 8.0. The analysis was performed on 9/3/3 (FOV/batches/cover glasses) per conditions. Statistical significance was evaluated using one-way ANOVA.

### Analysis of HumAfFt-NPM-BT1 fluorescence

To evaluate the ability of BT1-loaded NPM-HumAfFt complex to binding different form of Tau protein, differentiated DIV 30 neurons were incubated with 150 nM of BT1-loaded NPM-HumAfFt complex for 24 h at 37 °C in 5% CO_2_. Subsequently, cells were fixed with 4% paraformaldehyde (PFA, Sigma Aldrich) for 15 min at room temperature. The immunostaining was performed incubating fixed neurons with the primary antibodies HT7 (1:1000 mouse, MN1000, Invitrogen), AT8 (1:200 mouse, MN1020, Invitrogen) and T22 (1:200 rabbit, ABN454, Merck) overnight at 4 °C. The day after, the cells were incubated with secondary antibody Goat anti-rabbit or anti-mouse Alexa Fluor™ plus 647 (1:750, ThermoFisher Scientific) for 1 h at room temperature. Hoechst was used to stain nuclei. Fluorescence images of 2048 × 2048 pixels (6.5 µM/pixel) were acquired with oil objective 60X/NA 1.42 (Olympus) in stack with z-step of 0.2 µm, on an Olympus iX73 microscope equipped with an X-Light V3 spinning disc head (CrestOptics), an LDI laser illuminator (89 North), a PRIME cMOS camera and a MetaMorph software (Molecular Devices). Fluorescence images of the probe BT1 co-stained with the antibodies AT8, T22, and HT7 were analyzed through a custom code developed in the MATLAB environment. Maximum Z-projections of image stacks were first subjected to a pre-processing step consisting of the following operations: Gaussian filtering (sigma = 3 pixels), background removal, contrast enhancement, and H-minima transformation. More precisely, the Gaussian filter was used to smooth the images and reduce the noise, the background level was identified with a histogram shape-based method beyond the peak of the intensity distribution, the top 0.01% of the signal was saturated to enhance images contrast, and H-minima transform was additionally applied for further denoise. Furthermore, a mask of the cell body and neurite structure was extracted from the antibody image and applied to each channel to exclude unwanted signals. To obtain this mask, antibody images were binarized using a low threshold. Binarized images were skeletonized and separated regions underwent morphological dilation using a linear structuring element. If a dilated segment intersected with other segments, it was retained; otherwise, it was discarded. This procedure enables a robust reconstruction of the structure of the cytoskeleton, which when combined with a mask of the cell body (obtained with a global thresholding and size filtering), generates the desired mask. Pre-processed images were normalized and binarized to select meaningful pixels with a global thresholding method. To take into account the high variability of the signal distribution among images, a Matlab Guided User Interface was developed to adjust the preprocessing parameters and the signal selection, eliminating unspecific signals accurately. Finally, to quantify the colocalization of the probe BT1 with the antibodies AT8, T22 and HT7 we evaluated both Pearson’s correlation coefficient (PC) and Manders’ overlap coefficients (M1 and M2). While PC measures pixel intensity spatial correlations among pre-processed images of BT1 and antibodies, M1 (resp. M2) quantifies the co-occurrence of BT1 and antibodies as the fraction of the antibody (resp. probe) signal coinciding with the probe (resp. antibody) signal in the binarized images.

### Statistical data analysis

Statistical analysis, as well as the creation of graphs and plots, was conducted using GraphPad Prism 9–10 (GraphPad Software) and MATLAB 2016b (MathWorks). To assess the normality of our data sets, we employed the Shapiro–Wilk normality test. In cases where the data did not follow a normal distribution, we performed statistical significance analysis using the two-sided non-parametric Mann–Whitney test (MW test, *P* = 0.05). For all other cases, unless otherwise stated, we employed the ANOVA test (*P* = 0.05), and data sets are presented as mean values accompanied by the standard error of the mean (s.e.m.).

## Results

### Aggregation-induced quenching and poor solubility affects BT1 emission fluorescence

**Figure 1:**
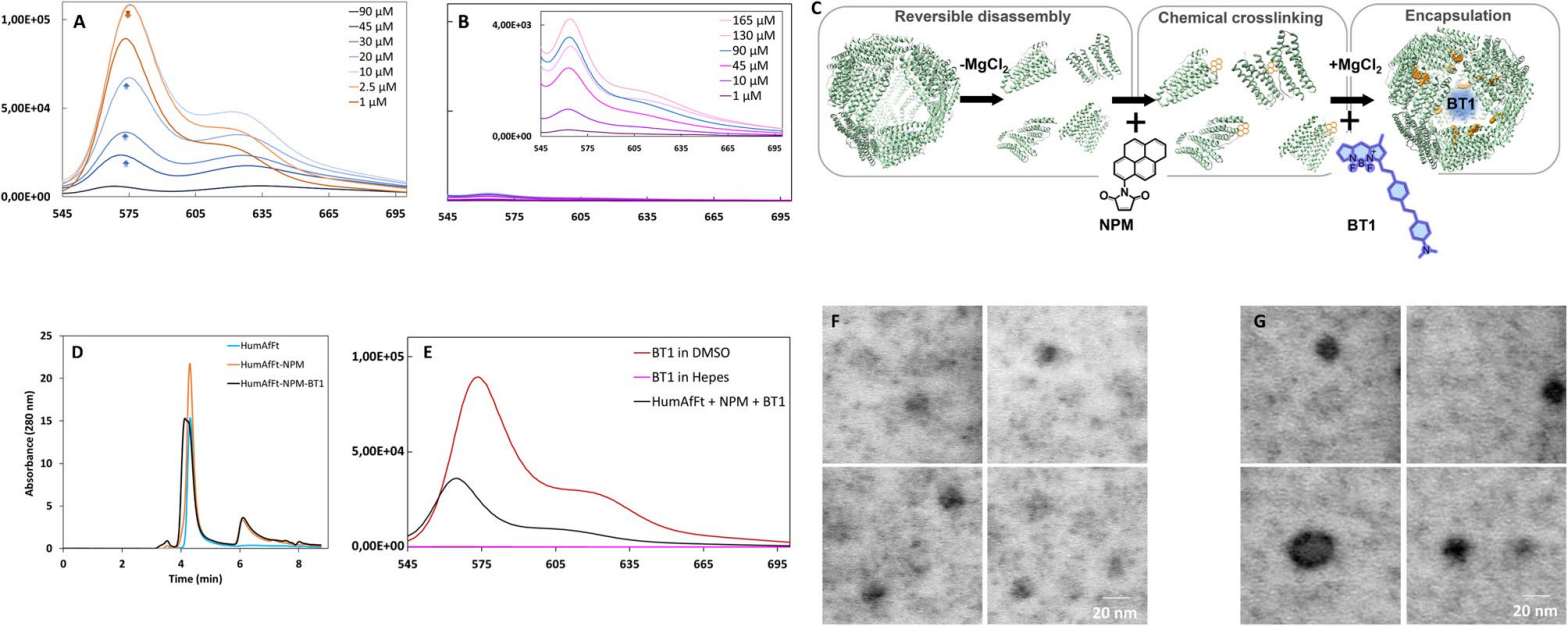
Characterization of successful BT1 encapsulation in ferritin nanoparticles. (**A**, **B**) Representative fluorescence spectra of BT1 showing the influence of polar solvents on the BT1 fluorescence: (**A**) Variations of fluorescence intensities as a function of BT1 concentration in 100% DMSO and (**B**) 20 mM Hepes pH 7.4: a closed-up view is included for clarity. (**C**) Illustrative drawing of the pyrene-based functionalization and BT1 encapsulation within ferritin nanocage. HumAfFt here is shown as green helices. NPM is displayed in orange sticks and covalently linked to C54 per monomer. BT1 structure is highlighted in blue and, for simplicity, represented as a blue sphere inside the nanocage. (**D**) Representative chromatograms showing HPLC analysis of HumAfFt (CTRL) in cyano, NPM-HumAfFt in orange and BT1-loaded NPM-HumAfFt in black (n = 3 preparations). (**E**) Fluorescence spectra of BT1 in NPM-HumAfFt in 20 mM Hepes, pH 7.4 is shown in green. Note the enhancement of fluorescence intensity induced by BT1 encapsulation. For comparison, 1 μM BT1 in 100% DMSO is shown in red and 1 μM BT1 dissolved in 20 mM Hepes pH 7.4 in magenta. NPM-HumAfFt does not show any absorbance in the observed range. All the spectra were measured in the 545–700 nm range (λ_ex_ = 520 nm; Ex. bandwidth = 5 nm; Em. bandwidth = 5 nm) and performed on 6 preparations. Representative STEM images of the HumAfFt (CTRL) and (**F**) BT1-loaded NPM-HumAfFt. Scale bars = 20 nm (n = 25 particles, 6 images, 2 samples for each condition). (**G**).

BT1 is a BODIPY-based compound functionalized with a highly conjugated system ending with an aliphatic amine conferring high hydrophobicity to the compound. Though BT1 is soluble in polar solvents, including DMSO, up to 10 mM concentration, it is poorly soluble in aqueous solutions (soluble at 300 μM), as often reported for BODIPY-based fluorophores^45^. The effect of solubility was studied by comparison of the fluorescence emission spectra in DMSO and in physiologic solutions (20 mM Hepes, pH = 7.4), as reported in Fig. 1A,B, respectively. The emission profile of BT1 diluted in DMSO in a range from 5 to 90 μM, displayed a decrease in fluorescence intensity upon increase in concentration, due to aggregation-induced quenching (AIQ) phenomenon^46^. AIQ was reverted at concentrations lower than 5 μM (2.5 μM, dark orange line, Fig. 1A). In contrast, BT1 first dissolved in 100% DMSO and then diluted in 20 mM Hepes pH 7.4, displayed very low fluorescence intensity with respect to 100% DMSO within a range from 1 to 230 μM. As shown in the onset in Fig. 1B, the fluorescence intensity increased as the concentration increased, and this correlation was almost linear ranging from 1 to 130 μM. Exceeding this limit concentration, an inversion occurred with the decreasing of the intensity shown as a dark blue line at 165 μM of Fig. 1A. The inversion of the trend is typical of AIQ^47^. Accordingly, Rayleigh scattering as a function of BT1 concentration in the aqueous solution revealed dispersed particles with higher colloid size and morphology (Fig. 1B).

### BT1 loaded in ferritin nanocages preserves its photochemical properties

In order to improve biocompatibility and address issues related to targeted delivery, BT1 was entrapped into HumAfFt-based nanocages. Ferritin protein nanocages display unique architecture, exceptional biocompatibility, and high functionalization capabilities^48^. Furthermore, ferritin nanocages can be uploaded by cells expressing the Transferrin Receptor 1, also known as CD71, which is expressed in many cell types^49^. However, preliminary attempts to incorporate the BT1 probe into the HumAfFt were unsuccessful due to the high hydrophobicity of the molecule (data not shown). Therefore, to obtain a rapid and feasible insertion of the BT1 probe, the internal cavity of HumAfFt was functionalized by chemical crosslinking to enhance hydrophobic interaction with the BODIPY core. In agreement with experimental conditions previously described^50^, HumAfFt was site-selectively labeled with multiple N-substituted maleimide compounds bearing different functionalities, such as N-(1-Pyrenyl)maleimide (NPM), 6-maleimidohexanoic acid and 1,6-Bis(maleimido)hexane, on a topologically selected C54 cysteine residue per monomer inside the protein cavity, as outlined in the scheme in Fig. 1C.

To favor the complete functionalization by thiol-reactive compounds, the HumAfFt was disassembled by removing MgCl_2_, taking advantage of its unique assembly–disassembly properties and shifting the equilibrium toward the dimeric form of the protein. The preferred maleimide-based compound, the N-(1-Pyrenyl)maleimide (NPM), was added in excess and the successful reaction was confirmed by MALDI-TOF mass spectrometry analysis. The obtained molecular weight of 20,565 Da for pyrene-labeled HumAfFt agreed with the predicted one (Figure S2A–B).

Ultimately, BT1 in excess was added and the encapsulation was favored by adding 50 mM MgCl_2_ which restored the 82,57% of the closed conformation of the ferritin nanocage as calculated by HPLC analysis (Fig. 1D). The assembly and dimensions of the newly formed nanoparticle were further confirmed by dynamic light scattering analysis and STEM images. This examination revealed a final hydrodynamic diameter of 17.8 ± 0.1 nm compared to the control of 15.3 ± 0.4 nm, confirming that the functionalization with NPM of the nanocage does not impact the overall protein assembly as previously described^50^. After the encapsulation of BT1, an enlargement of nanoparticles was observed as indicated by STEM images with a final dimension of 22.1 ± 4.8 nm (n = 25 particles, 2 preparations) (Fig. 1F,G). Then, the emission intensity of BT1-loaded in NPM-HumAfFt was evaluated after the removal of the unloaded probe. As reported in Fig. 1E, the fluorescence profile of BT1 encapsulated in NPM-HumAfFt (black line) showed a negligible Rayleigh scattering as a confirmation of an improved BT1 solubility in aqueous solution coupled with a tenfold increase in fluorescence emission intensity as compared to free BT1 in solution at 1 μM in 20 mM Hepes. The final concentration of BT1 encapsulated within the ferritin nanoparticle was calculated by UV–Vis analysis after opening of the nanoparticle upon addition of 100% DMSO and 150 mM EDTA. Based on the calibration curve (Figure S1), the BT1 concentration was 9.9 µM, with a protein content of 1.6 µM (24-mer). This corresponds to an average of about 6.2 ± 0.3 BT1 molecules per ferritin nanoparticle, as evidenced by three independent experiments. Furthermore, BT1-loaded in NPM-HumAfFt retained full fluorescence ability within 1 month from the incorporation (data not shown). Hence, our findings indicate the essential role of covalent modification of ferritin internal surface with pyrenyl-based compounds in capturing hydrophobic molecules like the BT1 probe.

### BT1 loaded in ferritin nanocages preserves its ability to selectively bind tau fibrils

Once successfully incorporated in NPM-HumAfF, the ability of such BT1-loaded to bind to K18 fibrils was evaluated in time-course experiments by fluorescence emission spectroscopy. Firstly, a control was chosen, to validate the selectivity of the binding between the probe and the tau fibrils. BSA is known to form fibrils within 5 h when subjected to high temperatures^22,40^. After 24 h at such temperatures, BSA reaches extreme levels of fibrillation, transforming the solution in a gelatinous sample.Before carrying out the Hum-BT1 fluorescence experiment, the fibrillation status of BSA and K18 was confirmed and evaluated using a well-known fluorophore, thioflavin T (ThT) (Figure S3). BSA showed fibrillation immediately, after 30 min and increased slightly after 5 h, whilst K18 started showing fibrillation only after 3–4 days, increasing steadily after 7 days (Figure S3). Given these results, the main experiment was performed based on these fibrillation times.

**Figure 2:**
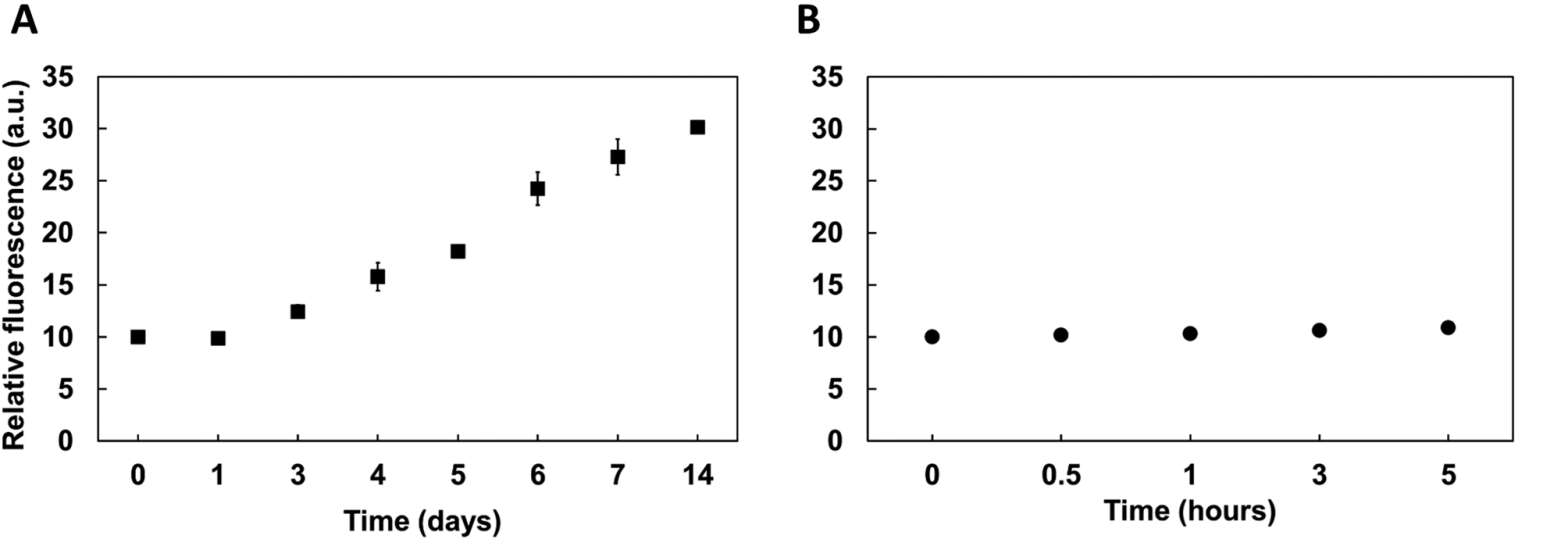
Time courses of fluorescence emission intensities of K18 tau protein and fibrillated BSA. Variation of fluorescence of BT1-loaded NPM-HumAfFt upon binding to β-sheet structures of (A) K18 tau protein before and after treatment with heparin at 37 °C (non-visible error bars are lower than 2%; n = 4 independent preparations) and (B) BSA protein before and after heating at 63 °C (non-visible error bars are lower than 2%; n = 4 independent preparations). All the spectra were measured using a λ_ex_ = 520 nm and λ_em_ = 565 nm.

Unfibrillated K18 protein (75 μM) and fibrillated K18 upon the addition of the aggregation promoter heparin (ratio 1:1) followed by incubation under mild agitation at 37 °C for different times, were analyzed after the addition of BT1-loaded NPM-HumAfFT to a final protein concentration of 1 μM. As already described, under these experimental conditions, elongated fibrils of about 200 nm appeared after 4 days, and the elongation process was complete at 7 days^22^. Our results revealed that the enclosed dye can be released from the nanocages and maintain the ability to interact with β-sheet structures of tau fibrils. This is evidenced by the increase in fluorescence intensity observed with the formation of K18 fibrils as depicted in Fig. 2A, where the maximum fluorescence (at λ_em_ = 565 nm) is plotted as a function of time. In addition, the selectivity of the binding between the probe and the tau fibrils was further validated exposing BT1-loaded NPM-HumAfFt to the β-sheet structures of aggregated BSA, and the fluorescence changes of the solution were followed^41,51^.

The experiment was then carried out using 75 μM BSA (CTRL) and fibrillated BSA upon heating at 63 °C after 0,5 h, 1 h, 3 h, and 5 h under mild agitation, conditions known to facilitate BSA fibril formation (Figure S3). The release of BT1 likely remains consistent in the presence of K18 fibrils compared to BSA fibrils. However, any significant increase in fluorescence intensity was not observed when excited at 520 nm, even with proteins exhibiting similar structural formations such as BSA as expected (Fig. 2B).

### Efficient uptake of BT1-loaded in NPM-HumAfFt from human iPSC-derived retinal cells

**Figure 3:**
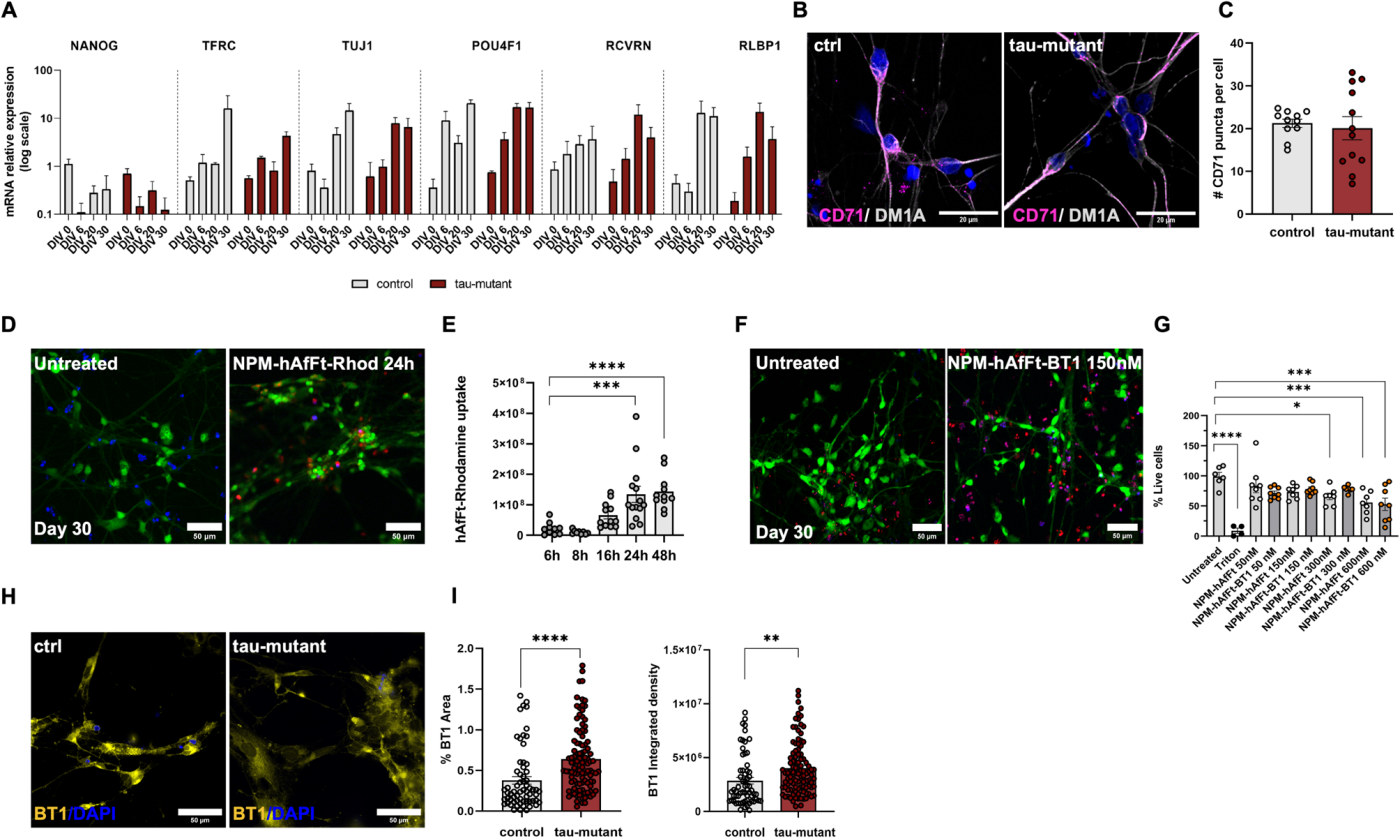
NPM-HumAfFt-BT1 Complex Demonstrates Successful Internalization in Retinal Neurons with Minimal Toxicity. (**A**) Bar chart showing the expression of retinal and neuronal transcripts in human control (gray bars) and tau-mutant (burgundy bars) iPSC-derived retinal cells during culture differentiation and maturation over a 30-day time course. Values are expressed as fold change normalized to DIV 0 of control cultures, set as 1 for each transcript. Retinal cell types are characterized by specific markers: NANOG for stem cells, TUJ1 for neuronal cells, POU4F1 (BRN3a) for retinal ganglion cells; RCVRN for photoreceptors; RLBP1 for Müller glia. Note that both control and tau-mutant retinal cultures express the transferrin receptor-CD71 (TFRC) (n = 15/3/3 fields of view/coverslips/independent cultures for both control and tau-mutant iPSCs; Student t-test or Mann Whitney ns). (**B**) Representative confocal images (maximum intensity projection 20 stacks, z = 0.2 µm) showing the expression of the transferrin receptor CD71 (magenta) in control and tau-mutant iPSC-derived retinal cultures at DIV 30. The cytoskeletal marker DM1A (gray) is used to identify neuronal cells and to create the mask used for the analysis. Scale bar: 20 µm. Nuclei were stained with HOECST (blue). (**C**) Bar chart showing the quantification of CD71 expression in control and tau-mutant hiPSC-derived retinal cultures at DIV 30 in control (light gray, n = 15/3/3 fields of view/coverslips/independent cultures for both control and tau-mutant iPSCs) and tau-mutant (burgundy, n = 15/3/3 fields of view/coverslips/independent cultures for both control and tau-mutant iPSCs Student t-test ns). (**D**) Representative confocal images of control hiPSC-derived retinal neurons at DIV 30 treated with 150 nM NPM-HumanAfFt-Rhodamine (red) for 24 h. Live cells were stained with Fluorescein Diacetate (FDA; green), nuclei were counterstained with HOECST in blue. The images depict a maximum intensity projection of a defined region of interest (20 stacks with a z-spacing of 0.2 µm). Scale bar: 50 µm. (**E**) Bar charts displaying the quantitative analysis of NPM-HumanAfFt-Rhodamine (150 nM) uptake by retinal neurons at DIV 30 at different time points (6, 8, 16, 24, and 48 h). The intensity of rhodamine fluorescence increases progressively over time, with statistically significant differences observed when compared to the 6-h treatment period (n = 15/3/3 fields of view/coverslips/independent cultures for both control and tau-mutant iPSCs, one-way ANOVA, ***p < 0.001, ****p < 0.0001). (F) Representative confocal images of control hiPSC-derived retinal neurons (DIV 30) treated with 150 nM NPM-HumanAfFt-BT1 complex for 24 h to evaluate BT1 cytotoxicity. Living cells are stained in green with FDA (green), dead cells are stained in red with Propidium iodide (red) and nuclei are stained in blue with HOECTS. Scale bar: 50 µm. Images show a maximum intensity projection of a region of interest (20 stacks, z = 0.2 µm). (**G**) Bar charts showing the dose–response effect on cell survival following 24-h exposure to NPM-HumanAfFt BT1 (dark gray bars with orange dots). Cells incubated with NPM-HumanAfFt (vehicle, light gray bars with white dots) are used as control. Cells treated with 10% triton for 4 min are used as control for dead cells (light gray bars with black dots). Untreated cells are used as positive control (light gray bars with white dots; n = 15/3/3 fields of view/coverslips/independent cultures for each condition). Significant differences are reported compared to the untreated condition (one-way ANOVA, *p < 0.05, **p < 0.01, ****p < 0.0001). (**H**) Representative confocal images illustrating the internalization of the NPM-HumAfFt-BT1 complex (yellow) within living retinal neurons at DIV 30 (control and tau-mutant). Nuclei were counterstained with HOECST in blue. The images represent a maximum intensity projection of a region of interest, composed of 16 stacks with a z-spacing of 0.2 µm. Scale bar: 50 µm. (**I**) Bar charts showing the quantification of the area covered by BT1 signal (left) and the integrated density analysis of BT1 signal (right) in DIV 30 control and tau-mutant retinal neurons. (n = 15/3/3 fields of view/coverslips/independent cultures for both control and tau-mutant iPSCs; one-way ANOVA, **p < 0.01, ****p < 0.0001).

Two isogenic human iPSC lines, designated as iPSC28 (control) and IVS10 + 16 (tau-mutant), were employed as a proof of concept to assess, in a humanized in vitro model, the efficacy of detecting pathological tau forms using BT1-loaded NPM-HumAfFt in the retina of neurodegenerative disease patients. Specifically, the iPSC line IVS10 + 16 (tau-mutant) was selected since it harbors the intronic 10 + 16 MAPT mutation associated with FTD. This mutation, which enhances the inclusion of exon 10, disrupts the balance between the 3R and 4R tau isoforms, ultimately leading to an increase in pathological tau variants^52^. Both iPSC lines were differentiated into retinal cultures according to a previously published protocol^42,43,53^ with minor adaptations (Figure S4A). The multi-stage differentiation process spanned approximately 30 days (from DIV0 to DIV 30) and employed small molecules to establish a uniform and well-distributed 2D network of retinal cells. To validate the successful differentiation of both the control and tau-mutant lines into retinal cell populations, a time-course quantification of key retinal cell molecular markers was conducted using real-time PCR (Fig. 3A). As expected, there was a substantial increase in transcripts associated with neurons (TUJ1), photoreceptors (RCVRN), Müller cells (RLBP1), and ganglion cells (POU4F1) over the course of the culture period. Additionally, as maturation progressed, the NANOG transcript levels decreased, favoring the expression of retinal neuronal markers^42,43^. The maturation of retinal cells was further confirmed at the protein level through confocal immunofluorescence analysis at DIV 30, which confirmed the presence of BRN3A, TUJ1, and MAP2 markers in both control and tau-mutant cultures (Figure S4B,C). Furthermore, to validate the potential ability of retinal cells to uptake HumAfFt, the presence of the transferrin receptor CD71^54^, which is known to be functionally expressed in the rat and mouse retina^36–39^, was evaluated in human retinal cells. Real-time PCR analysis (Fig. 3A) and quantitative immunofluorescence (Fig. 3B) demonstrated that CD71 was similarly expressed at DIV 30 in both control and tau-mutant cultures (Fig. 3C). Based on these observations, the functional ferritin uptake by human iPSC-derived retinal cells was tested in a time-lapse experiment^35,51^. Figure 3D illustrates the temporal progression of rhodamine-ferritin conjugate uptake (at a concentration of 150 nM) in control DIV 30 human iPSC-derived retinal neurons used as proof of retinal internalization of ferritin nanocages. The internalization process was assessed by monitoring the rhodamine fluorescence within viable cells and subsequently quantified at different time intervals (6, 8, 16, 24, and 48 h; Fig. 3D and Figure S5). The intensity of rhodamine fluorescence exhibited a gradual increase over time, with a plateau observed at the 24-h mark, indicating that 24 h represents the optimal incubation period (Fig. 3D, E) to be used for NPM-HumAfFt-BT1 complex upload.

In evaluating the safety profile of BT1 for retinal cells, we conducted a live-dead assay on iPSC-derived control retinal cells at DIV 30. The experiment involved treating these cells with varying concentrations of the NPM-HumAfFt-BT1 complex, ranging from 50 to 600 nM. We used NPM-HumAfFt as a comparator and Triton (10% for 4 min) as a positive control for cell death induction. After 24 h of exposure, the cells were stained with Fluorescein Diacetate (FDA) for live cells and Propidium Iodide for dead cells, ensuring the imaging wavelengths avoided interference with BT1 fluorescence (Fig. 3F and Figure S5). Crucially, our findings revealed that the NPM-HumAfFt-BT1 complex displayed a favorable safety profile at concentrations up to 150 nM. It was at higher concentrations, specifically 300 nM and 600 nM, that significant cytotoxic effects were observed (Fig. 3G). This result underscores the potential of using a 150 nM dose of the NPM-HumAfFt-BT1 complex as a safe and effective means for detecting pathological tau in living retinal cells. This concentration strikes a balance, offering efficacy in tau detection while minimizing cellular toxicity.

The uptake of the BT1-loaded in NPM-HumAfFt (150 nM) by retinal cells was then assessed in both control and tau-mutant retinal cultures at DIV 30 following a 24-h incubation period (Fig. 3H). Quantitative analysis of BT1 fluorescence, conducted using confocal acquisitions at an excitation wavelength of 520 nm, demonstrated the successful uptake of BT1 (Fig. 3I). The higher BT1 signal observed in tau-mutant cultures (Fig. 3I) was consistent with the expected increase in BT1 fluorescence when bound to tau pathological forms^22^ (see Fig. 2) that are expected to be enhanced in cultures differentiated from iPSC harboring the intronic 10 + 16 MAPT mutation associated with FTD^52,55–58^.

### Enhanced specificity of the BT1-loaded in NPM-HumAfFt for pathological tau forms in human retinal cells

To thoroughly investigate the effectiveness of BT1 in identifying diverse forms of tau protein, both control and tau-mutant retinal cultures were treated with the BT1 compound encapsulated in the NPM-HumAfFt delivery system at a concentration of 150 nM, maintained over a 24-h period at 37 °C. Post-treatment, the retinal cultures underwent a comprehensive immunolabeling procedure. This involved the use of three specific antibodies: HT7, AT8, and T22, each targeting different tau protein forms—total tau, phosphorylated tau (p-tau) at Ser202/Ser205, and oligomeric tau (o-tau), respectively. By leveraging the delivery capabilities of the ferritin nanocages, this method allowed for a thorough evaluation of the NPM-HumAfFt-BT1 complex's effectiveness in distinguishing and detecting various tau protein forms in living human retinal cells.

The confocal acquired images (λ_ex_ = 520 nm, λ_em_ = 560/640 nm) were analyzed using a custom MATLAB code that simultaneously conducted background correction, detected signals associated with antibodies, and quantified fluorescence intensity and channel colocalization (see Methods section). The co-localization of BT1 with total tau signal (HT7) confirmed the ability of BT1 to bind tau protein (Figure S6).

**Figure 4:**
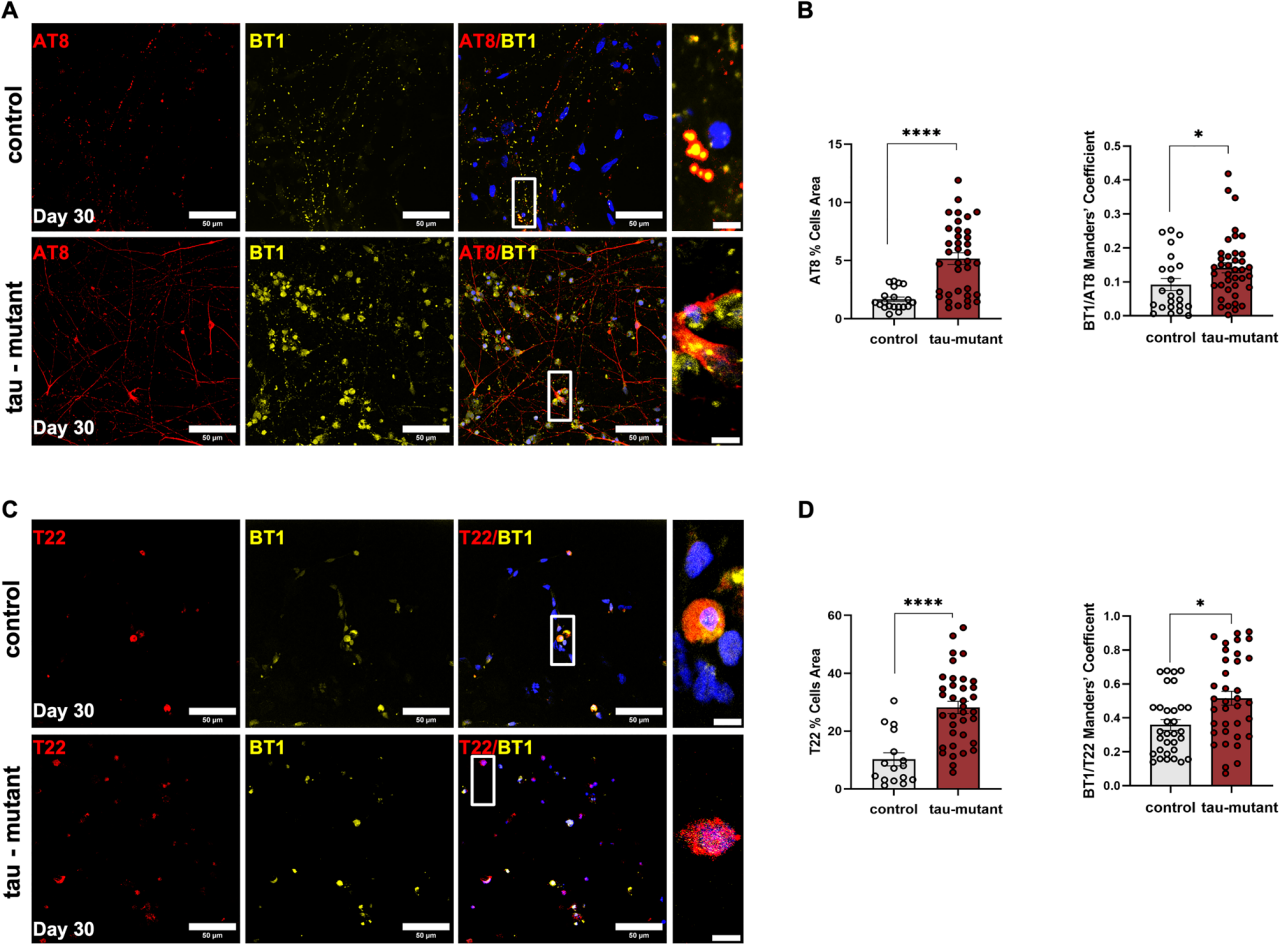
NPM-HumAfFt-BT1 Nanocages Enable Accurate Detection of p-tau and o-tau in Retinal Neurons (**A**) Representative confocal images of control (top) and tau-mutant (bottom) iPSC-derived retinal neurons (at DIV 30) treated with 150 nM NPM-HumanAfFt-BT1 complex for 24 h, then fixed and immunostained for anti PHF-tau Ser202/Thr205 antibody (AT8). Images show a maximum intensity projection of a region of interest. Scale bar: 50 µm. Zoomed images reveal the cytoplasmic co-localization of BT1 (yellow) and AT8 (red). HOECST was used to stain nuclei (blue). Scale bar: 5 µm. (**B**) Bar charts showing the quantification of the area covered by AT8 (left) and the Manders co-localization coefficient of BT1 and AT8 (right) in control (grey) and tau-mutant (burgundy) retinal neurons (n = 15/3/3 fields of view/coverslips/independent cultures for both control and tau-mutant iPSCs, one-way ANOVA *p < 0.05,****p < 0.0001). (**C**) Representative confocal images of control (top) and tau- mutant (bottom) iPSC-derived retinal neurons (at DIV 30) treated with 150 nM NPM-HumanAfFt-BT1 complex for 24 h, then fixed and immunostained for anti o-tau antibody (T22). Images show a maximum intensity projection of a region of interest. Scale bar: 50 µm. Zoomed images reveal the cytoplasmic co-localization of BT1 (yellow) and T22 (burgundy). HOECST was used to stain nuclei (blue). Scale bar: 5 µm. (**D**) Bar charts showing the quantification of the area covered by T22 staining (left) and the Manders co-localization coefficient of BT1 and T22 signals (right) in control (gray) and tau-mutant (burgundy) retinal neurons. (n = 15/3/3 fields of view/coverslips/independent cultures for both control and tau-mutant iPSCs, one-way ANOVA *p < 0.05, ****p < 0.0001).

As depicted in Fig. 4A, AT8 staining showed that tau-mutant retinal cells exhibited elevated p-tau expression compared to control cells (Fig. 4B, left). The efficient binding of the p-tau form by BT1-loaded in NPM-HumAfFt was demonstrated through the colocalization of AT8 and BT1, quantified using Mander's coefficient (Fig. 4B, right).

Moreover, T22 staining revealed an increased presence of o-tau in tau-mutant retinal cultures at DIV 30 compared to control cultures. The NPM-HumAfFt-BT1 complex effectively detected the heightened expression of o-tau, as depicted in Fig. 4C,D.

In line with previous research, we found similar results in control human iPSC-derived retinal cultures treated with 50 nM okadaic acid (OA) for 2 h. This treatment is a recognized method for inducing tau hyperphosphorylation and triggering neuronal degeneration^22,26^. We evaluated the effects of OA by analyzing the fluorescence signal intensity of both physiological and pathological tau proteins using AT8, T22, and HT7 antibodies (Figure S7A–D).

Remarkably, these findings align well with previously reported data from our lab and others, where BODIPY-based tau ligands were used in similar experiments involving iPSC-derived neurons^22^ and neuroblastoma cell lines^26^. A key discovery in our current research is the effective internalization of the BT1 compound, delivered through the NPM-HumAfFt system, into living human retinal cells. This uptake likely occurs via the CD71 receptor. Moreover, we observed that BT1, once inside the cells, specifically binds to pathological tau forms related to FTD^52,55–58^, while maintaining a level of cytotoxicity within acceptable limits.

## Discussion

In this study, we have developed an innovative ferritin nanocage-based system for the delivery of BODIPY-based tau fluorophores, aimed at detecting tau proteins in living human cells. This approach is crucial for the study and the diagnosis of neurodegenerative diseases like AD and FTD. Our key findings include: (1) The NPM-HumAfFt protein nanocages successfully address the solubility and emission challenges of the BT1 compound; (2) Once encapsulated, BT1 exhibits selective binding to fibrillated tau proteins; (3) These protein nanocages efficiently transport BT1 into living human retinal cells; (4) Encapsulated BT1 within NPM-HumAfFt nanocages demonstrates a high specificity in identifying pathological tau forms, coupled with low cytotoxicity, underscoring its potential for reliable diagnostic applications.

As widely reported, near infrared bodipy-based fluorophores, such as BT1, have gained considerable attention in diagnostic applications for their excellent photochemical luminescence^59,60^. However, they are not without potential drawbacks since they are poorly soluble in biological environments raising concerns about efficient biodistribution and in vivo delivery. Indeed, the behavior of BT1 fluorescence emission in aqueous solutions indicates that BT1 solubility remains insufficient for achieving optimal emission intensity for in vitro applications. Moreover, the spectroscopic properties of BT1, particularly its fluorescence emission, are significantly altered by the aggregation-induced quenching phenomenon. In polar solvents, fluorescence intensity decreases with increasing concentration in DMSO due to specific interactions in the nearest solvation shell, such as hydrogen bonds and π-stacking^45^. In aqueous solutions, this effect is coupled with strong insolubility, leading to very low fluorescence intensity of the probe and impossibility to use it for in vitro testing and analysis. To address these issues, BT1 was successfully entrapped into ferritin nanocages agents^35,61–65^. Ferritin nanocages are widely employed for delivering various types of molecules, including chemotherapeutics, siRNA, drugs, and imaging agents and can be engineered through gene-editing or chemical functionalization to enhance their targeting capabilities, loading capacity, and bioavailability^66–68^. However, the incorporation of hydrophobic molecules inside ferritin is challenging and modification of native ferritin with hydrophobic amino acid sequences by selected mutagenesis has been reported as mandatory^69^. We highlighted the significance of chemically crosslinking HumAfFt internal cavity with NPM to enhance hydrophobic interactions with the BT1 compound. This not only provides a valuable alternative to genetic engineering but also holds great potential for various applications in the biomedical field, particularly considering the widespread use of hydrophobic drugs in cancer treatment and diagnosis.

We confirmed the successful encapsulation of BT1 into the NPM-HumAfFt complex through HPLC and STEM analyses, which showed an increase in nanoparticle dimensions while preserving their integrity. We hypothesize that the increase in nanoparticle volume results from hydrophobic interactions between NPM and BT1. Remarkably, BT1 entrapped into the NPM-modified ferritin nanocages preserved its photochemical properties, demonstrated by a significant increase in fluorescence emission intensity in aqueous solutions compared to free BT1. This encapsulation also retained full fluorescence ability over a sustained period, indicating stability. In addition, the BT1-loaded NPM-HumAfFt complex demonstrated selective binding to tau fibrils. This specificity was fundamental, given that tau protein anomalies are hallmarks of various neurodegenerative disorders.

Using fluorescence emission spectroscopy, we quantified the BT1 content within each NPM-HumAfFt complex, establishing a ratio of 6:1 molecules. This ratio suggests that the functionalized nanocage not only enhances BT1 solubility in physiological fluids but also optimizes its targeted delivery. Additionally, we demonstrated that BT1 loaded in NPM-HumAfFt binds specifically to K18 tau fibrils, whereas it shows no affinity for BSA fibrils, despite both having the capability to form β-sheet structures. This specificity of binding to tau fibrils over BSA, as previously discussed in Soloperto et al.^22^.Moreover, our data indicate a successful and efficient uptake of BT1-loaded NPM-HumAfFt by human iPSC-derived retinal cells^42,43,53^ This was demonstrated using two isogenic human iPSC lines, including a tau-mutant line harboring a MAPT mutation associated with FTD, providing a model to assess the efficacy of detecting pathological tau forms^52^.

The differentiation and maturation of these iPSC lines into retinal cells were confirmed using a variety of molecular markers, including the expression of the transferrin receptor 1 (CD71), which is essential for ferritin uptake. Our cell cultures exhibited a diverse array of retinal cells, including photoreceptors, bipolar cells, amacrine cells, ganglion cells, and Müller glia. At day 30, these cells showed widespread expression of CD71. This observation is consistent with findings from rodent models^36–38^ and is supported by data from the Human Protein Atlas (https://www.proteinatlas.org/ENSG00000072274-TFRC/brain/retina). Such data support the expression of CD71 in retinal ganglion and other inner retinal cells, which are key targets for addressing the tau tangles associated with neurodegenerative diseases.

We report a gradual increase in the uptake of the NPM-HumAfFt-BT1 complex over time, with minimal cytotoxicity observed when used at a concentration of 150 nM. This concentration was identified as optimal for detecting pathological tau forms in living retinal cells while maintaining cell viability. The internalization process, confirmed through confocal imaging and quantitative analysis, indicated that the BT1-loaded NPM-HumAfFt was successfully taken up by the retinal cells. Furthermore, the observed elevation in BT1 fluorescence within tau-mutant cultures aligns with anticipations of augmented BT1 fluorescence upon binding to pathological tau forms^22^ This phenomenon is particularly notable in cultures derived from iPSCs containing the intronic, FTD related, 10 + 16 MAPT mutation^52,55–58^.

Strikingly, the NPM-HumAfFt delivery system efficiently carries and releases the BT1 compound within the cellular environment, facilitating the selective binding to various tau forms. We hypothesize that the release of BT1 occurs through a mechanism that mimics the natural behavior of human ferritin within early endosomes, as described in previous studies^49,70^.

The efficacy of BT1 binding to pathological tau forms was evidenced by the successful co-localization of BT1 with the staining obtained with specific antibodies (HT7, AT8, and T22) targeting total tau, phosphorylated tau (p-tau), and oligomeric tau (o-tau), respectively, especially BT1 staining was consistent with the elevated expression of p-tau and o-tau in tau-mutant cultures compared to controls, highlights the system's specificity and effectiveness. Notably, the use of okadaic acid to induce tau hyperphosphorylation further corroborated the ability of BT1 to distinguish between physiological and pathological tau, aligning with the expected outcomes for tauopathies such as AD and FTD^17,22–24,26,64,66–69^.

This correlation underscores the specificity and sensitivity of our approach in detecting pathological tau, highlighting its potential utility in identifying and studying tauopathies at a cellular level.

Comparatively, our results mirror findings from previous studies that employed BODIPY-based tau ligands for the detection of tau in iPSC-derived neurons and neuroblastoma cell lines^22,26^. Notably, our study advances the field by addressing the solubility and delivery challenges associated with BODIPY compounds through the novel use of ferritin nanocages. The effective internalization of BT1, facilitated by the CD71 receptor, not only ensures the compound's delivery into retinal cells but also maintains the probe's photochemical properties and specificity towards pathological tau forms, with minimal cytotoxicity observed, underscoring its potential as a diagnostic tool for neurodegenerative diseases, particularly those associated with tauopathies like FTD.

## Conclusions

The recent development of tau specific fluorophores has moved the retinal imaging approach to Alzheimer diagnostics center stage. The properties of a novel generation of fluorophores, based on BODIPY scaffold (including the recently identified lead compound, BT1), comprise a very high affinity for tau oligomers coupled with optimal fluorescent properties, well suited for retinal imaging. Nevertheless, poor water solubility together with unexplored biodistribution and delivery properties represent crucial drawbacks for in vitro and in vivo utilization of these compounds. The body of experimental results here presented provides novel insights in the context of BT1 targeted delivery. In particular, efficient BT1-loading into NPM-HumAfFt protein-based nanocages resulted in enhanced solubility while preserving the photochemical characteristics of BT1. Moreover, BT1-loaded NPM-HumAfFt was shown to be efficiently and selectively incorporated in human retinal cells and to bind tau fibrils with high affinity and selectivity, revealing its potential as a tool for the investigation of tauopathies. Furthermore, minimal toxicity on human iPSC-derived retinal cultures was observed. The immunolabeling and colocalization experiments underscored the specific interaction of BT1 with pathological tau forms, suggesting its viability for pathological tau detection and bioimaging applications. Additionally, our research demonstrated the effectiveness of BT1-loaded NPM-HumAfFt in human retinal cells, particularly in tau-mutant cultures, corroborating its applicability for in vivo use and pathological tau detection. In conclusion, this study provides a comprehensive examination of the challenges and opportunities associated with BT1 and related BODIPY compounds. By addressing its solubility constraints and highlighting its potential in tauopathy-related applications, we have contributed to the understanding of BT1 as a valuable tool for further research in the field of tau monitoring in neurodegenerative diseases.

It is important to note that while the use of ferritin nanocages has addressed a primary challenge in delivering BT1 to retinal cells, numerous hurdles remain. These include the efficacy of reaching retinal cells in vivo, overcoming the inner limiting membrane, and identifying non-invasive and clinically viable administration methods. These challenges are common across all nanoparticle retinal delivery systems, such as micelles, dendrimers, and liposomes, which require administration strategies ranging from invasive approaches like intravitreal or subretinal injections, to non-invasive methods like intranasal delivery already used to target brain cells^71,72^. Addressing these issues will be crucial for advancing the application of nanoparticle-based delivery systems in retinal diagnostics and therapy.

### Supplementary Information

Supplementary Information.

## Data Availability

The datasets used and/or analysed during the current study are available from the corresponding author on reasonable request. For the analysis of imaging data, we utilized custom code developed in MathWorks Matlab (version 2016b), and this code is available upon request.

## Abbreviations

AD: Alzheimer’s disease
FTD: Frontotemporal Dementia
MRI: Magnetic resonance imaging
PET: Positron emission tomography
CSF: Cerebrospinal fluid
FP: Fundus photography
OCT: Optical coherence tomography
OCT‐A: Optical coherence tomography
FLIO: Fluorescence lifetime imaging ophthalmoscopy
HIS: Hyperspectral imaging
Aβ: Beta-amyloid
hiPSCs: Human-induced pluripotent stem cells
HumAfFt: Humanized archaeoglobus ferritin
SEC: Size-exclusion chromatography
HPLC: High performance liquid chromatography
STEM: Scanning transmission electron microscopy
NPM: *N*-(1-pyrenyl)maleimide
TCEP: Tris(2-carboxyethyl)phosphine hydrochloride
DLS: Dynamic light scattering
BSA: Bovine serum albumin
ThT: Thioflavin T
Au: Arbitrary units
DIV: Days in vitro
RT-qPCR: Real-time quantitative polymerase chain reaction
PFA: Paraformaldehyde
NA: Numericalb aperture
FDA: Fluorescein diacetate
PI: Propidium iodide
NES: Normal extracellular solution
PC: Pearson’s correlation coefficient
M1 and M2: Manders’ overlap coefficients
AIQ: Aggregation-induced quenching
NPM: *N*-(1-pyrenyl)maleimide
MALDI-TOF: Matrix-assisted laser desorption/ionization coupled to time-of-flight
OA: Okadaic acid
p-tau: Phosphorylated tau
o-tau: Oligomeric tau
TfR1: Transferrin receptor protein 1
MgCl2: Magnesium chloride
mM: Millimolar
rpm: Revolutions per minute
°C: Celsius degree
pH: Potential of hydrogen
UV–Vis: Ultraviolet–visible
ε: Molar extinction coefficient
mg: Milligram
g: Grams
µm: Micrometre
RT: Room temperature
DLS: Dynamic light scattering
mW: Molecular weight
nm: Nanometer
PBS: Phosphate buffered saline
TCEP: (Tris(2-carboxyethyl)phosphine
mm^2^: Square millimeter
Pen-Strep: Penicillin-streptomycin
NEAA: Non-essential amino acids
w/o: Without
PLO: Poly-l-ornithine
ml: Milliliter
ANOVA: Analysis of variance
NaCl: Sodium chloride
KCl: Potassium chloride
CaCl_2_: Calcium chloride
LDI: Laser diode illuminator
MW: Mann–Whitney

## Code availability

For the analysis of imaging data, we utilized custom code developed in MathWorks Matlab (version 2016b), and this code is available upon request.

## References

[1] Huang LK, Kuan YC, Lin HW, Hu CJ (2023). Clinical trials of new drugs for Alzheimer disease: A 2020–2023 update. J. Biomed. Sci..

[2] Cummings J, Zhou Y, Lee G, Zhong K, Fonseca J, Cheng F (2023). Alzheimer’s disease drug development pipeline: 2023. Alzheimer’s Dementia Transl. Res. Clin. Interv..

[3] Self WK, Holtzman DM (2023). Emerging diagnostics and therapeutics for Alzheimer disease. Nat. Med..

[4] Wang YTT, Rosa-Neto P, Gauthier S (2023). Advanced brain imaging for the diagnosis of Alzheimer disease. Curr. Opin. Neurol..

[5] Ossenkoppele R, Pichet Binette A, Groot C, Smith R, Strandberg O, Palmqvist S (2022). Amyloid and tau PET-positive cognitively unimpaired individuals are at high risk for future cognitive decline. Nat. Med..

[6] Pizzarelli R, Pediconi N, Di Angelantonio S (2020). Molecular imaging of tau protein: new insights and future directions. Front. Mol. Neurosci..

[7] Hansson O, Edelmayer RM, Boxer AL, Carrillo MC, Mielke MM, Rabinovici GD (2022). The Alzheimer’s Association Appropriate Use Recommendations for Blood Biomarkers in Alzheimer’s Disease. Alzheimer’s and Dementia.

[8] Lim JKH, Li QX, He Z, Vingrys AJ, Wong VHY, Currier N (2016). The Eye as a biomarker for Alzheimer’s Disease. Frontiers in Neuroscience.

[9] Hart NJ, Koronyo Y, Black KL, Koronyo-Hamaoui M (2016). Ocular indicators of Alzheimer’s: exploring disease in the retina. Acta Neuropathologica.

[10] Snyder PJ, Alber J, Alt C, Bain LJ, Bouma BE, Bouwman FH (2021). Retinal imaging in Alzheimer’s and neurodegenerative diseases. Alzheimer’s Dementia.

[11] López-Cuenca I, Salobrar-García E, Elvira-Hurtado L, Fernández-Albarral JA, Sánchez-Puebla L, Salazar JJ (2021). The value of oct and octa as potential biomarkers for preclinical Alzheimer’s disease: A review study. Life.

[12] Ashraf G, McGuinness M, Khan MA, Obtinalla C, Hadoux X, van Wijngaarden P (2023). Retinal imaging biomarkers of Alzheimer’s disease: A systematic review and meta-analysis of studies using brain amyloid beta status for case definition. Alzheimer’s Dementia Diagn. Assess. Dis. Monit..

[13] Hussain A, Sheikh Z, Subramanian M (2023). The eye as a diagnostic tool for Alzheimer’s disease. Life.

[14] Pediconi N, Gigante Y, Cama S, Pitea M, Mautone L, Ruocco G (2023). Retinal fingerprints of ALS in patients: Ganglion cell apoptosis and TDP-43/p62 misplacement. Front. Aging Neurosci..

[15] Gupta VB, Chitranshi N, den Haan J, Mirzaei M, You Y, Lim JK (2021). Retinal changes in Alzheimer’s disease—Integrated prospects of imaging, functional and molecular advances. Progress Retinal Eye Res..

[16] Grimaldi A, Pediconi N, Oieni F, Pizzarelli R, Rosito M, Giubettini M (2019). Neuroinflammatory processes, A1 astrocyte activation and protein aggregation in the retina of Alzheimer’s disease patients, possible biomarkers for early diagnosis. Front. Neurosci..

[17] Verwilst P, Kim HS, Kim S, Kang C, Kim JS (2018). Shedding light on tau protein aggregation: The progress in developing highly selective fluorophores. Chem. Soc. Rev..

[18] Bajad NG, Kumar A, Singh SK (2023). Recent Advances in the Development of Near-Infrared Fluorescent Probes for the in Vivo Brain Imaging of Amyloid-β Species in Alzheimer’s Disease.

[19] Liu Y, Zhuang D, Wang J, Huang H, Li R, Wu C (2022). Recent advances in small molecular near-infrared fluorescence probes for a targeted diagnosis of the Alzheimer disease. Analyst. Roy. Soc. Chem..

[20] Yu X, Li C, Wang B, Ding X, Wang N, Xing B (2024). Protein-mediated fluorescent probes for bioimaging and biosensing: From fundamentals to applications. TrAC Trends Anal. Chem..

[21] Seo Y, Park KS, Ha T, Kim MK, Hwang YJ, Lee J (2016). A smart near-infrared fluorescence probe for selective detection of tau fibrils in Alzheimer’s disease. ACS Chem. Neurosci..

[22] Soloperto A, Quaglio D, Baiocco P, Romeo I, Mori M, Ardini M (2022). Rational design and synthesis of a novel BODIPY-based probe for selective imaging of tau tangles in human iPSC-derived cortical neurons. Sci. Rep..

[23] Akasaka T, Watanabe H, Ono M (2023). In vivo near-infrared fluorescence imaging selective for soluble amyloid β aggregates using y-shaped BODIPY derivative. J. Med. Chem..

[24] Ma S, Chen G, Xu J, Liu Y, Li G, Chen T (2021). Current strategies for the development of fluorescence-based molecular probes for visualizing the enzymes and proteins associated with Alzheimer’s disease. Coord. Chem. Rev..

[25] Verwilst P, Kim HR, Seo J, Sohn NW, Cha SY, Kim Y (2017). Rational design of in vivo tau tangle-selective near-infrared fluorophores: expanding the BODIPY universe. J. Am. Chem. Soc..

[26] Li Y, Tian C, Xie T, Zhang QL, Liu J, Yan XX (2023). Hydroxyethyl-modified cycloheptatriene-BODIPY derivatives as specific tau imaging probes. ACS Med. Chem. Lett..

[27] Teppang KL, Zhao Q, Yang J (2023). Development of fluorophores for the detection of oligomeric aggregates of amyloidogenic proteins found in neurodegenerative diseases. Front. Chem..

[28] D’Antoni C, Mautone L, Sanchini C, Tondo L, Grassmann G, Cidonio G (2023). Unlocking neural function with 3D in vitro models: A technical review of self-assembled, guided, and bioprinted brain organoids and their applications in the study of neurodevelopmental and neurodegenerative disorders. Int. J. Mol. Sci..

[29] Cordella F, Brighi C, Soloperto A, Di Angelantonio S (2022). Stem cell-based 3D brain organoids for mimicking, investigating, and challenging Alzheimer’s diseases. Neural Regen. Res..

[30] Brighi C, Cordella F, Chiriatti L, Soloperto A, Di Angelantonio S (2020). Retinal and brain organoids: Bridging the gap between in vivo physiology and in vitro micro-physiology for the study of Alzheimer’s diseases. Front. Neurosci..

[31] Brighi C, Salaris F, Soloperto A, Cordella F, Ghirga S, de Turris V (2021). Novel fragile X syndrome 2D and 3D brain models based on human isogenic FMRP-KO iPSCs. Cell Death Dis..

[32] Steimberg N, Bertero A, Chiono V, Dell’Era P, Di Angelantonio S, Hartung T (2020). iPS, organoids and 3d models as advanced tools for in vitro toxicology. ALTEX.

[33] Park J, Wetzel I, Marriott I, Dréau D, D’Avanzo C, Kim DY (2018). A 3D human triculture system modeling neurodegeneration and neuroinflammation in Alzheimer’s disease. Nat. Neurosci..

[34] Lin YT, Seo J, Gao F, Feldman HM, Wen HL, Penney J (2018). APOE4 causes widespread molecular and cellular alterations associated with Alzheimer’s disease phenotypes in human iPSC-derived brain cell types. Neuron.

[35] De Turris V, Cardoso Trabuco M, Peruzzi G, Boffi A, Testi C, Vallone B (2017). Humanized archaeal ferritin as a tool for cell targeted delivery. Nanoscale.

[36] Yefimova MG, Jeanny JC, Guillonneau X, Keller N, Nguyen-Legros J, Sergeant C (2000). Iron, ferritin, transferrin, and transferrin receptor in the adult rat retina. Invest Ophthalmol. Vis. Sci..

[37] Kumar P, Nag TC, Jha KA, Dey SK, Kathpalia P, Maurya M (2017). Experimental oral iron administration: Histological investigations and expressions of iron handling proteins in rat retina with aging. Toxicology.

[38] Picard, E., Jonet, L., Sergeant, C., Vesvres, M.H., Behar-Cohen, F., Courtois, Y., *et al*. Overexpressed or intraperitoneally injected human transferrin prevents photoreceptor degeneration in rd10 mice (2010). Available from: http://www.molvis.org/molvis/v16/a280 PMC3002967 21179240

[39] Baksi S, Tripathi AK, Singh N (2016). Alpha-synuclein modulates retinal iron homeostasis by facilitating the uptake of transferrin-bound iron: Implications for visual manifestations of Parkinson’s disease. Free Radic. Biol. Med..

[40] Dasgupta M, Kishore N (2017). Selective inhibition of aggregation/fibrillation of bovine serum albumin by osmolytes: Mechanistic and energetics insights. PLoS One.

[41] Vetri V, D’Amico M, Foderà V, Leone M, Ponzoni A, Sberveglieri G (2011). Bovine Serum Albumin protofibril-like aggregates formation: Solo but not simple mechanism. Arch. Biochem. Biophys..

[42] Sluch VM, Chamling X, Liu MM, Berlinicke CA, Cheng J, Mitchell KL (2017). Enhanced stem cell differentiation and immunopurification of genome engineered human retinal ganglion cells. Stem Cells Transl. Med..

[43] Sluch VM, Davis CHO, Ranganathan V, Kerr JM, Krick K, Martin R (2015). Differentiation of human ESCs to retinal ganglion cells using a CRISPR engineered reporter cell line. Sci. Rep..

[44] Schneider CA, Rasband WS, Eliceiri KW (2012). NIH Image to ImageJ: 25 years of image analysis HHS public access. Nat. Methods.

[45] Antina LA, Kalyagin AA, Ksenofontov AA, Pavelyev RS, Lodochnikova OA, Islamov DR (2021). Effect of polar protic solvents on the photophysical properties of bis(BODIPY) dyes. J. Mol. Liq..

[46] Wang H, Li Q, Alam P, Bai H, Bhalla V, Bryce MR (2023). Aggregation-induced emission (AIE), life and health. ACS Nano.

[47] Zhang K, Liu J, Zhang Y, Fan J, Wang CK, Lin L (2019). Theoretical study of the mechanism of aggregation-caused quenching in near-infrared thermally activated delayed fluorescence molecules: Hydrogen-bond effect. J. Phys. Chem. C.

[48] Zhang C, Zhang X, Zhao G (2020). Ferritin nanocage: A versatile nanocarrier utilized in the field of food, nutrition, and medicine. Nanomaterials.

[49] Li L, Fang CJ, Ryan JC, Niemi EC, Lebrón JA, Björkman PJ (2010). Binding and uptake of H-ferritin are mediated by human transferrin receptor-1. Proc. Natl. Acad. Sci. U. S. A..

[50] Benni I, Trabuco MC, Di Stasio E, Arcovito A, Boffi A, Malatesta F (2018). Excimer based fluorescent pyrene-ferritin conjugate for protein oligomerization studies and imaging in living cells. RSC Adv..

[51] Militello V, Casarino C, Emanuele A, Giostra A, Pullara F, Leone M (2004). Aggregation kinetics of bovine serum albumin studied by FTIR spectroscopy and light scattering. Biophys Chem..

[52] Verheyen A, Diels A, Reumers J, Van Hoorde K, Van den Wyngaert I, van Outryve DC (2018). Genetically engineered iPSC-derived FTDP-17 MAPT neurons display mutation-specific neurodegenerative and neurodevelopmental phenotypes. Stem Cell Rep..

[53] Ferraro G, Gigante Y, Pitea M, Mautone L, Ruocco G, Di Angelantonio S (2023). A model eye for fluorescent characterization of retinal cultures and tissues. Sci. Rep..

[54] Montemiglio LC, Testi C, Ceci P, Falvo E, Pitea M, Savino C (2019). Cryo-EM structure of the human ferritin–transferrin receptor 1 complex. Nat. Commun..

[55] Kopach O, Esteras N, Wray S, Abramov AY, Rusakov DA (2021). Genetically engineered MAPT 10+16 mutation causes pathophysiological excitability of human iPSC-derived neurons related to 4R tau-induced dementia. Cell Death Dis..

[56] Kopach O, Esteras N, Wray S, Rusakov DA, Abramov AY (2020). Maturation and phenotype of pathophysiological neuronal excitability of human cells in tau-related dementia. J. Cell Sci..

[57] Setó-Salvia N, Esteras N, de Silva R, de Pablo-Fernandez E, Arber C, Toomey CE (2022). Elevated 4R-tau in astrocytes from asymptomatic carriers of the MAPT 10+16 intronic mutation. J. Cell Mol. Med..

[58] Shi Y, Zhang W, Yang Y, Murzin AG, Falcon B, Kotecha A (2021). Structure-based classification of tauopathies. Nature.

[59] Huang Q, Xie J, Liu Y, Zhou A, Li J (2017). Detecting the formation and transformation of oligomers during insulin fibrillation by a dendrimer conjugated with aggregation-induced emission molecule. Bioconjug. Chem..

[60] Xu L, Gao H, Zhan W, Deng Y, Liu X, Jiang Q (2023). Dual aggregations of a near-infrared aggregation-induced emission luminogen for enhanced imaging of Alzheimer’s disease. J. Am. Chem. Soc..

[61] Mainini F, Bonizzi A, Sevieri M, Sitia L, Truffi M, Corsi F (2021). Protein-based nanoparticles for the imaging and treatment of solid tumors: The case of ferritin nanocages, a narrative review. Pharmaceutics.

[62] Pediconi N, Ghirga F, Del Plato C, Peruzzi G, Athanassopoulos CM, Mori M (2021). Design and synthesis of piperazine-based compounds conjugated to humanized ferritin as delivery system of siRNA in cancer cells. Bioconjug. Chem..

[63] Pagani F, Testi C, Grimaldi A, Corsi G, Cortese B, Basilico B (2020). Dimethyl fumarate reduces microglia functional response to tissue damage and favors brain iron homeostasis. Neuroscience.

[64] Incocciati A, Kubeš J, Piacentini R, Cappelletti C, Botta S, Bertuccini L (2023). Hydrophobicity-enhanced ferritin nanoparticles for efficient encapsulation and targeted delivery of hydrophobic drugs to tumor cells. Protein Sci..

[65] Macone A, Masciarelli S, Palombarini F, Quaglio D, Boffi A, Trabuco MC (2019). Ferritin nanovehicle for targeted delivery of cytochrome C to cancer cells. Sci. Rep..

[66] Sevieri M, Pinori M, Chesi A, Bonizzi A, Sitia L, Truffi M (2023). Novel bioengineering strategies to improve bioavailability and in vivo circulation of h-ferritin nanocages by surface functionalization. ACS Omega.

[67] Gu C, Zhang T, Lv C, Liu Y, Wang Y, Zhao G (2020). His-mediated reversible self-assembly of ferritin nanocages through two different switches for encapsulation of cargo molecules. ACS Nano.

[68] Falvo E, Arcovito A, Conti G, Cipolla G, Pitea M, Morea V (2020). Engineered human nanoferritin bearing the drug genz-644282 for cancer therapy. Pharmaceutics.

[69] Wang YH, Jian ML, Chen PJ, Tsou JC, Truong LP, Wang YS (2021). Ferritin conjugates with multiple clickable amino acids encoded by C-terminal engineered Pyrrolysyl-tRNA synthetase. Front. Chem..

[70] Crielaard BJ, Lammers T, Rivella S (2017). Targeting iron metabolism in drug discovery and delivery. Nat. Rev. Drug Discov..

[71] Tawfik M, Chen F, Goldberg JL, Sabel BA (2022). Nanomedicine and drug delivery to the retina: Current status and implications for gene therapy. Naunyn Schmiedebergs Arch. Pharmacol..

[72] Marrocco F, Falvo E, Mosca L, Tisci G, Arcovito A, Reccagni A (2024). Nose-to-brain selective drug delivery to glioma via ferritin-based nanovectors reduces tumor growth and improves survival rate. Cell Death Dis..

