# Supplementary material for: Ferritin nanocage-enabled detection of pathological tau in living human retinal cells
Source: Sci Rep. 2024 May 21;14:11533. doi: 10.1038/s41598-024-62188-8 (PMC11109090; doi:10.1038/s41598-024-62188-8)
Supplement: Supplementary file 1 — Supplementary Information. [file 41598_2024_62188_MOESM1_ESM.pdf]

Supplementary Information

Ferritin Nanocage-Enabled Detection of Pathological Tau in Living Human Retinal Cells

Lorenzo Barolo, Ylenia Gigante, Lorenza Mautone, Silvia Ghirg, Alessandro Soloperto, Alessandra Giorgi, Francesca Ghirga, Martina Pitea, Alessio Incocciati, Francesco Mura, Giancarlo Ruocco, Alberto Boffi, Paola Baiocco, Silvia Di Angelantonio

Table of Contents:

1. Calibration curve for BT1 spectroscopic analysis
2. Preparation of functionalized HumAfFt with NPM
3. ThT fluorescence of BSA and K18
4. Retinal differentiation protocol and retinal cell characterization
5. Uptake and toxicity tests
6. NPM-HumAfFt BT1 detection of total tau in retinal cells
7. NPM-HumanAfFt-BT1 complex detection of different tau forms in Okadaic acid treated retinal cultures

Calibration curve for determination of BT1 molar extinction coefficient in 100% DMSO

The molar extinction coefficient ε for BT1 at 593 nm was calculated from a calibration curve. The BT1 powder was weighed and resuspended in 100% DMSO, reaching a final concentration of 4.4 mM. This stock solution was then diluted to collect multiple points (10, 20, 25, 50, 75, 90, 100, 125, 150, and 200 µM). The absorbance was measured at 593 nm in a 0.1 cm cuvette.

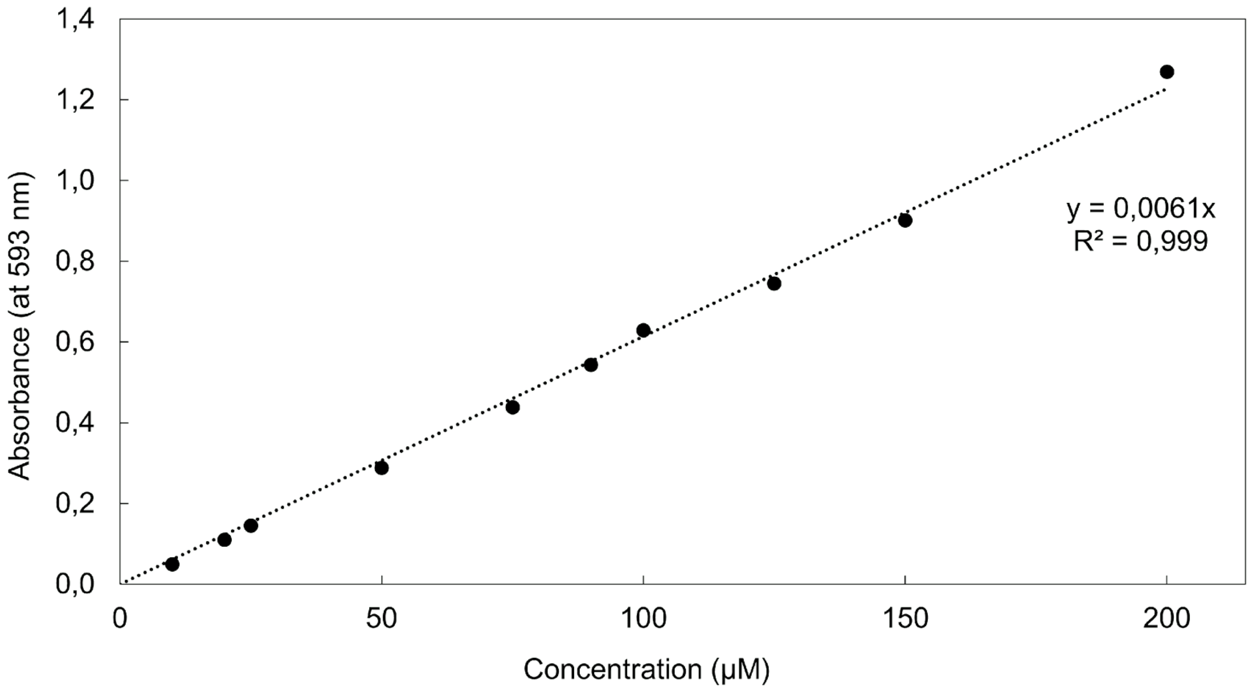

Supplementary Figure S1. Calibration curve absorbance vs concentration of BT1 resuspended in 100% DMSO. The molar extinction coefficient was calculated using a linear trendline to determine the equation of the line. The R-squared value of 0.999 proved the quality of the measurement. The obtained ε_{593nm} value was 61000 M^{-1} cm^{-1}.

NPM-HumAfFt conjugation analysis via mass spectrometry

The successful insertion of the maleimide in the internal cavity of the HumAfFt was confirmed by a mass spectrometry analysis. A sample of HumAfFt control and one linked with NPM were diluted to 1 μg/μL. The protein samples were analyzed by an UltrafleXtreme MALDI ToF/ToF, Bruker (Bruker, Bremen DE), equipped with a smartbeam-II laser, in linear and positive mode. The samples were mixed with the sinapinic acid matrix and solubilized in an aqueous solution containing 70% acetonitrile and 0.1% trifluoroacetic acid (TFA) in a ratio of 1:5 (sample: matrix).

The bioconjugation yields confirmed about 80% of cysteines modified by measuring the molar ratio between the maleimide and protein concentrations through UV-vis measurements.

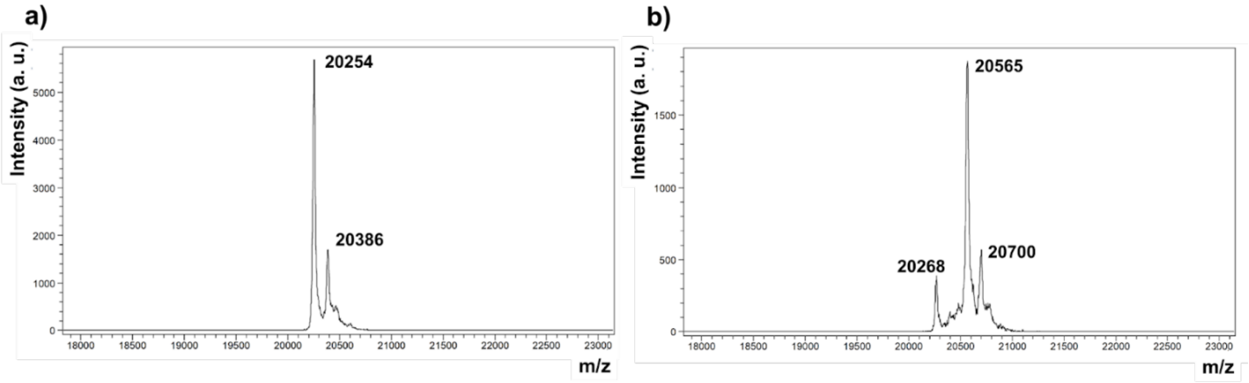

Supplementary Figure S2. Bioconjugation of NPM to HumAfFt. Mass Spectrometry analysis of A) HumAfFt (1 mg/mL) with a molecular mass of HumAfFt without and with the first methionine, 20254 Da and 20386 Da, respectively B) NPM-labelled HumAfFt with a molecular mass of 20565 Da and with (20700 Da) the first methionine. The peak at 20268 Da is related to HumAfFt unreacted.

ThT fluorescence analysis of fibrillated BSA and K18

K18 75 µM was kept at 37°C and different time points were selected for fluorescence analysis (0, 2, 5, 24, 48, 72, 96, 168 hours). K18 protein did not show any variation in fluorescence intensity before 72 hours (3 days). A 3-fold increase, compared to the control, was visible after 96 hours (4 days), reaching a 5-fold value after 168 hours (7 days). BSA 75 µM was kept at 63°C and different time points were selected for fluorescence analysis (0, 0.5, 1, 2, 3, 4, 5, 20 and 24 hours). It was not possible to evaluate samples over 24 hours because after that period of time under those conditions, the liquid BSA solution turned to gelatinous, and adding the fluorophore became impossible. BSA protein showed a 3-fold fluorescence intensity rise after 0.5 hours (30 min), steadily increasing up to 4-fold intensity after 5 hours. After 20 hours, the fluorescence intensity reached an intensity value 5-fold compared to the control. After 24 hours (1 day), this value remained stable, showing no increase, demonstrating that BSA had entered the lag-phase of fibrillation.

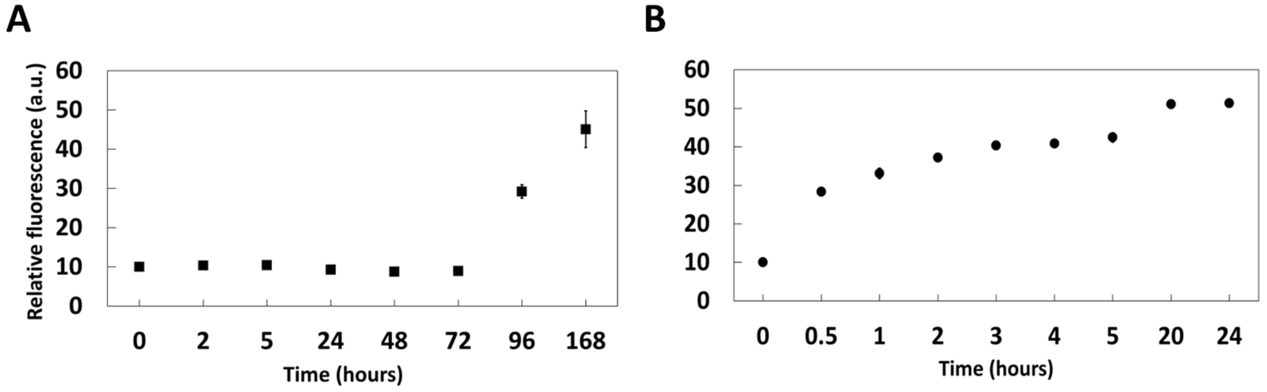

Supplementary Figure S3. Time courses of fluorescence emission intensities of K18 tau protein and fibrillated BSA. Variation of fluorescence of ThT upon binding to β-sheet structures of (A) K18 tau protein before and after treatment with heparin at 37°C (non-visible error bars are lower than 2%) and (B) BSA before and after heating at 63°C (non-visible error bars are lower than 2%). All the spectra were measured using a λ_{ex} = 430 nm and λ_{em} = 485 nm.

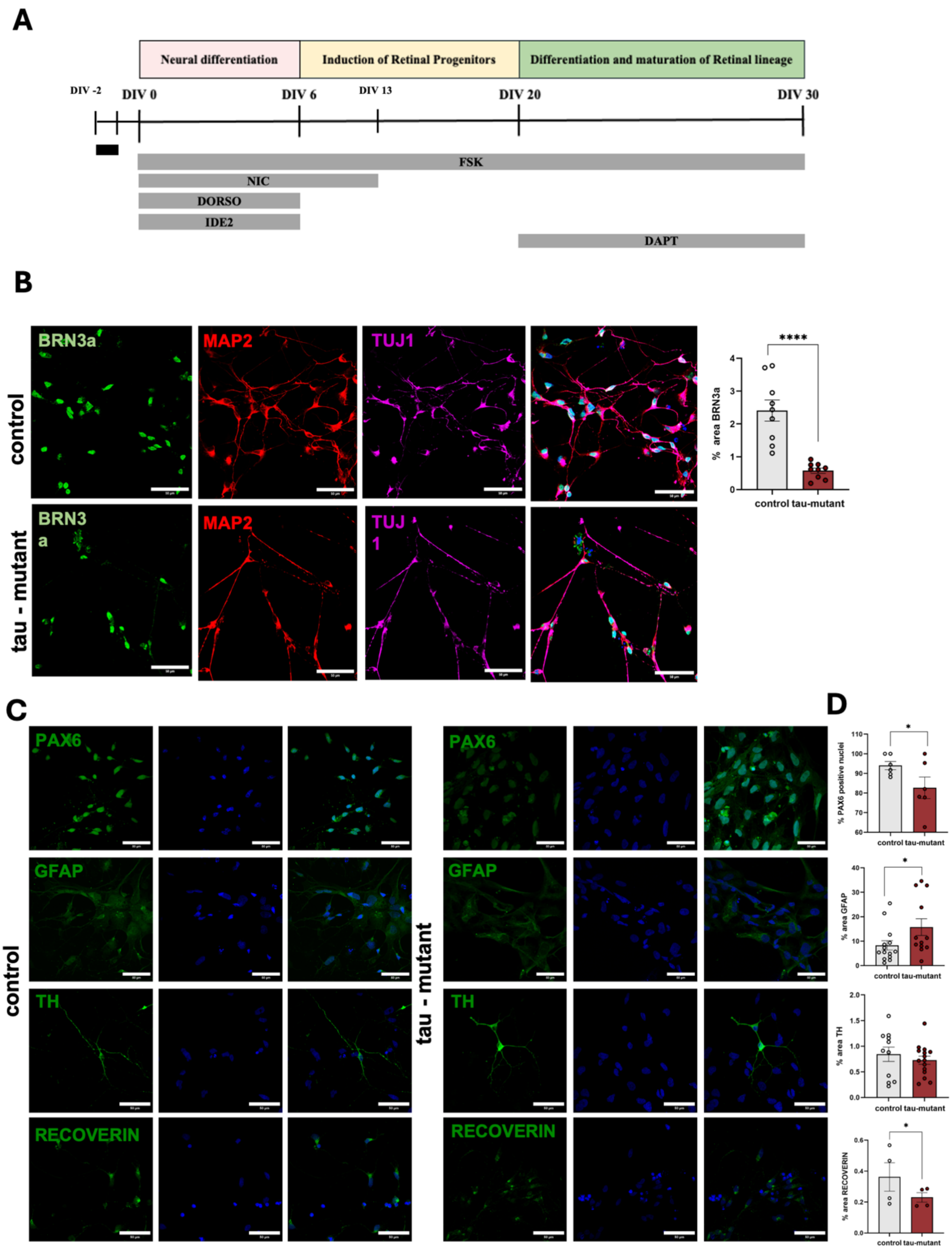

Supplementary Figure S4. A) Representative confocal images of DIV 30 retinal cultures differentiated from control (top) and tau-mutant (bottom) control human iPSC, stained for BRN3a (green), TUJ1 (magenta) and MAP2 (red). Scale bar: 50 µm. Nuclei were stained with HOECST (blue); **B)** Bar charts showing the quantification of the area covered by BRN3a staining in control (gray) and tau-mutant (burgundy) retinal neurons. (n= 9 FOV/3 batches, Unpaired t-test ****$p<0.0001$); **C)** Representative confocal images of DIV 30 retinal cultures differentiated from control (left) and tau-mutant (right) human iPSC, stained for PAX6, GFAP, TH, RECOVERIN (green). Scale bar: 50 µm. Nuclei were stained with HOECST (blue); **D)** Bar charts showing the quantification of the PAX6 positive cells in control (gray) and tau-mutant (burgundy) (n=6 FOV/3 batches, Unpaired t-test *$p<0.05$); Bar charts showing the area covered by GFAP staining (n=12 FOV/3 batches, Mann Whitney * $p<0.05$) TH (n=12 FOV/3 batches, Unpaired t-test, ns) RECOVERIN (n=4 FOV/3 batches, Unpaired t-test, ns) in control (gray) and tau-mutant (burgundy) retinal neurons.

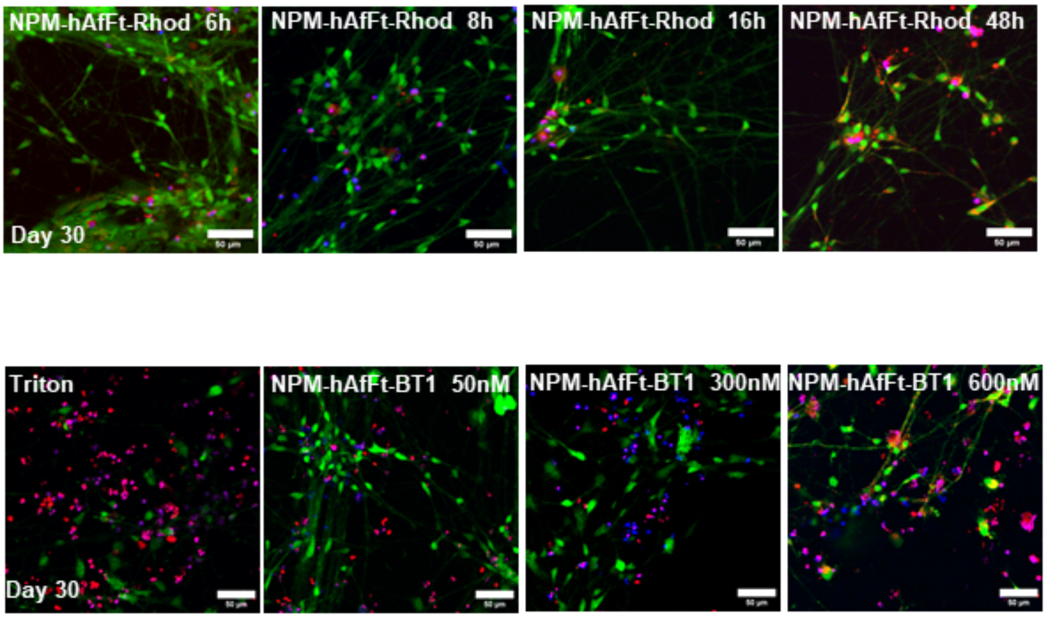

Supplementary Figure S5. Uptake and Low Toxicity of NPM-HumAfFt-BT1 Complex. Top. Representative confocal images of control human iPSC-derived retinal neurons at DIV 30 treated with 150 nM NPM-HumanAfFt-Rhodamine (red) to evaluate the nanocage uptake at 6h, 8h, 16h, 48h time intervals. Bottom. Representative confocal images of hiPSC-derived retinal neurons (DIV 30) treated with increased concentration of NPM-HumanAfFt-BT1 for 24 hours (50nM, 300nM, 600nM). Cells were stained with 10% triton for 4 minutes as control for dead cells.

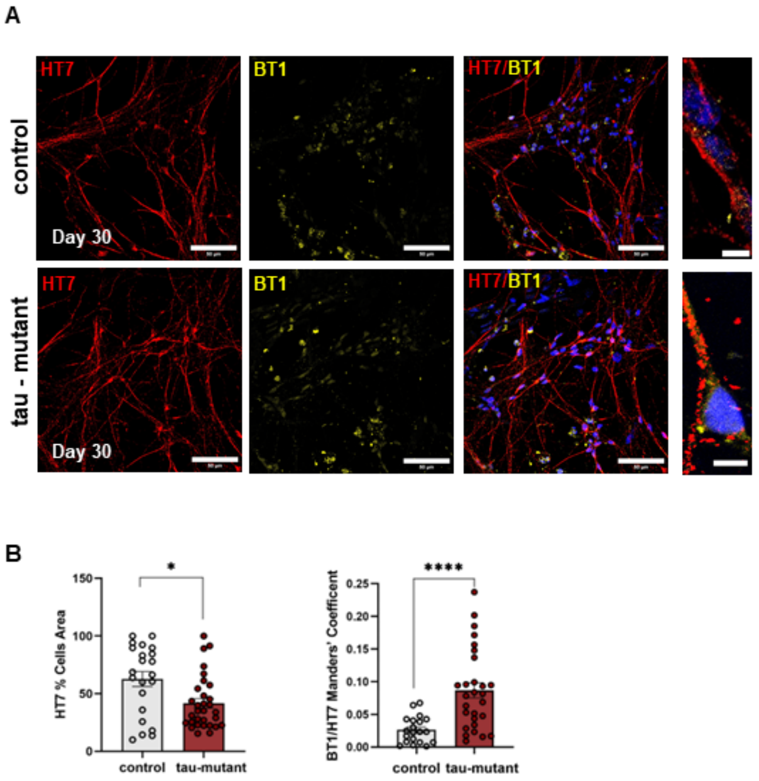

Supplementary Figure S6. NPM-HumAfFt-BT1 Nanocages Enable Accurate Detection of total tau in Retinal Neurons. (A) Representative confocal images of control (top) and tau-mutant (bottom) iPSC-derived retinal neurons (at DIV 30) treated with 150 nM of NPM-HumanAfFt-BT1 complex for 24 hours, then fixed and immunostained for anti total tau antibody (HT7). Images show a maximum intensity projection of a region of interest. Scale bar: 50 µm. Zoomed images reveal the cytoplasmic co-localization of BT1 (yellow) and HT7 (red). HOECST was used to stain nuclei (blue). Scale bar: 5 µm. (B) Left: Bar chart showing the quantification of the area covered by HT7 in control (gray bars) and tau-mutant (burgundy bars) retinal neurons. Right: Bar chart showing the Manders co-localization coefficient of BT1 and HT7 signals in control (gray bars) and tau-mutant (burgundy bars) retinal neurons. (n= 3, one-way ANOVA $*p<0.05$, $****p<0.0001$).

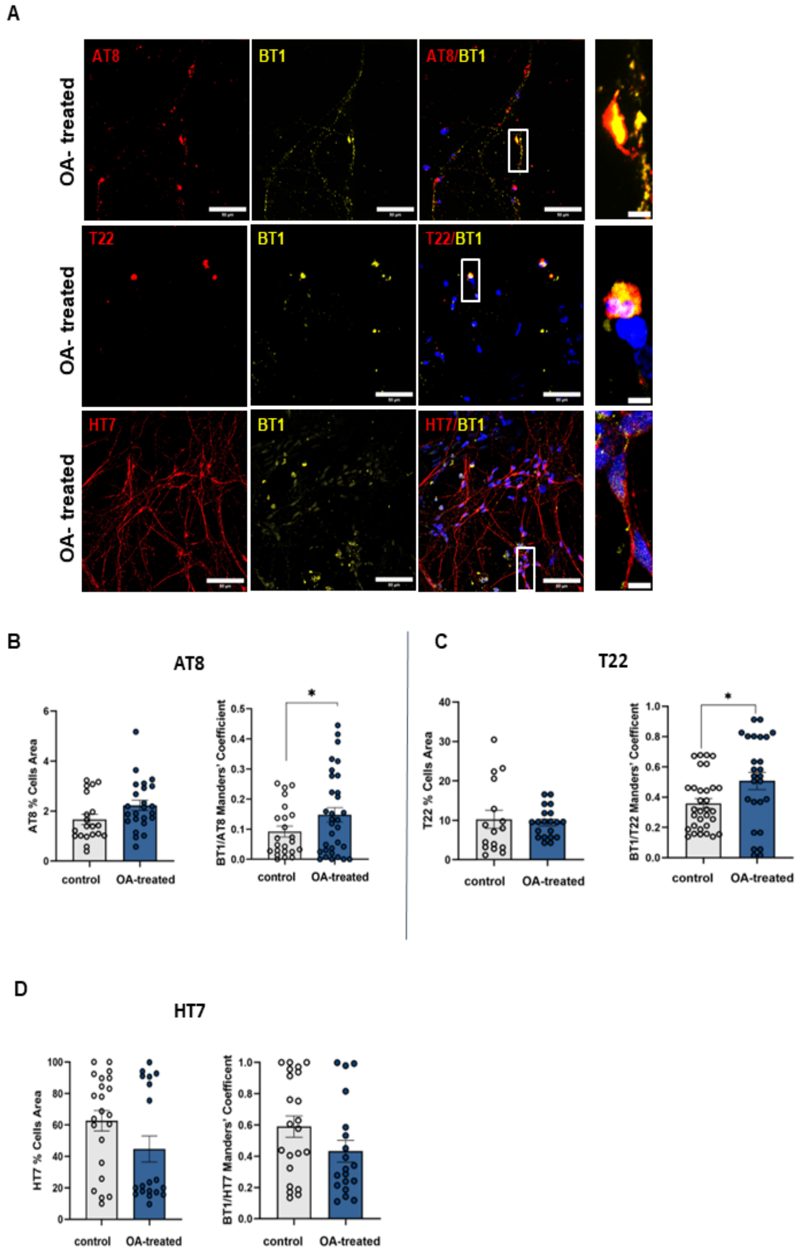

Supplementary Figure S7. NPM-HumanAfFt-BT1 complex bind Hyperphosphorylated and Oligomeric tau in Okadaic acid treated retinal neurons. (A) Representative confocal images of control iPSC-derived retinal neurons treated with 100 µM of Okadaic acid (OA) to induce tau hyperphosphorylation. Control and OA- treated cultures were incubated with 150 nM human NPM-HumAfFt-BT1 complex for 24 hours, fixed and immunostained for anti PHF-tau Ser202/Thr205 antibody (AT8), anti oligomeric-tau antibody (T22) and anti total tau antibody (HT7). Images show a maximum intensity projection of a region of interest. Scale bar: 50 µm. Zoomed images reveal the cytoplasmic co-localization of BT1 (yellow) with AT8 (red), HT7 (red), and nuclear co-localization with T22 (red). HOECST was used to stain nuclei (blue). Scale bar: 5 µm. (B) Left: Bar chart showing the quantification of the area covered by AT8 in control (gray bars) and OA-treated (blue bars) retinal neurons. Right: Bar chart showing the Manders co-localization coefficient of BT1 and AT8 signals in control (gray bars) and OA-treated (blue bars) retinal neurons. (n=3 batches, one-way ANOVA *p<0.05). (C) Left: Bar chart showing the quantification of the area covered by T22 in control (gray bars) and OA-treated (blue bars) retinal neurons. Right: Bar chart showing the Manders co-localization coefficient of BT1 and T22 signals in control (gray bars) and OA-treated (blue bars) retinal neurons. (n=3 batches one-way ANOVA *p<0.05). (D) Left: Bar chart showing the quantification of the area covered by HT7 in control (gray bars) and OA-treated (blue bars) retinal neurons. Right: Bar chart showing the Manders co-localization coefficient of BT1 and HT7 signals in control (gray bars) and OA-treated (blue bars) retinal neurons. (n=3 batches, one-way ANOVA ns).
